# Raman Analysis of RNA Nucleotide Strands From SARS‐CoV‐2 Variants and Subvariants: A Step Forward in the Definition of “Raman Genome”

**DOI:** 10.1002/advs.77679

**Published:** 2026-09-30

**Authors:** Giuseppe Pezzotti, Yoshiki Yasukochi, Gianluca Angiola, Takaharu Ueno, Saki Ikegami, Rintaro Okawa, Tetsuya Adachi, Wenliang Zhu, Osam Mazda, Alfio Grillo, Koichiro Higasa, Kazu Okuma

**Affiliations:** ^1^ Biomedical Engineering Center Kansai Medical University Hirakata Osaka Prefecture Japan; ^2^ International Center For Biomedical Industrial Promotion Kansai Medical University Hirakata Osaka Prefecture Japan; ^3^ Department of Immunology Graduate School of Medical Science Kyoto Prefectural University of Medicine Kyoto Kyoto Prefecture Japan; ^4^ Department of Orthopedic Surgery Tokyo Medical University Shinjuku‐ku Tokyo Prefecture Japan; ^5^ Department of Molecular Science and Nanosystems Ca’ Foscari University of Venice Venice Italy; ^6^ Biomarker Disease Laboratory IRCCS San Camillo Hospital Venice Lido Italy; ^7^ Department of Orthopaedic Surgery Graduate School of Medicine Mie University Tsu Mie Prefecture Japan; ^8^ Graduate School of Medicine Teikyo University Institute of Medical Mycology (TIMM) Teikyo University Itabashi‐ku Tokyo Prefecture Japan; ^9^ Department of Bacteriology Graduate School of Biomedical & Health Sciences Hiroshima University Hiroshima Hiroshima Prefecture Japan; ^10^ Laboratory For Technologies and Advanced Therapies (LTTA) Department of Medical Science University of Ferrara Ferrara Italy; ^11^ Emergency Department Fondazione Policlinico Universitario Agostino Gemelli IRCCS Roma Italy; ^12^ Department of Genome Analysis Institute of Biomedical Science Kansai Medical University Hirakata Osaka Prefecture Japan; ^13^ Department of Mathematical Science Politecnico di Torino Torino Italy; ^14^ Department of Microbiology Faculty of Medicine Kansai Medical University Hirakata Osaka Prefecture Japan; ^15^ Ceramic Physics Laboratory Kyoto Institute of Technology Kyoto Kyoto Prefecture Japan; ^16^ Department of Dental Medicine Graduate School of Medical Science Kyoto Prefectural University of Medicine Kyoto Kyoto Prefecture Japan

**Keywords:** genome, principal component analysis, Raman spectroscopy, RNA nucleotide strands, SARS‐CoV‐2, virus classification

## Abstract

Raman spectra of RNA nucleotide strands extracted from 20 SARS‐CoV‐2 strains, including the original Japanese isolate, Alpha, Beta, Gamma, Delta, Lambda, Theta, Mu, and 12 Omicron subvariants, were systematically collected, deconvoluted, and converted into Raman barcodes. The spectra exhibited common features associated with the RNA backbone, ribofuranose rings, and nitrogenous bases, reflecting the overall structural similarity of the viral RNAs. However, distinct differences were observed in spectral signatures related to β‐D‐ribofuranose vibrations, phosphate linkages, and RNA bases sensitive to secondary‐structure variations. Conventional principal component analysis provided only limited discrimination among variants and subvariants. In contrast, Raman barcodes captured subtle molecular‐scale structural differences and successfully distinguished all investigated strains according to their RNA secondary structures. The analysis identified vibrational markers associated with nucleotide chain length, backbone conformation, and ribofuranose dynamics. Several backbone‐ and base‐related signals exhibited hyperchromic behavior correlated with RNA secondary‐structure features, including hairpin content and stem length. These relationships may serve as indicators for the rapid detection of emerging variants. By encoding key spectral signatures, Raman barcodes provide a robust framework for accurate classification of SARS‐CoV‐2 strains based on the molecular architecture of their RNAs.

## Graphical abstract

Main Raman signals in RNA, related to helical backbone, ribofuranose ring, and nitrogenous bases, are affected by secondary structures that cause hyperchromic effects and significantly alter the Raman intensity.

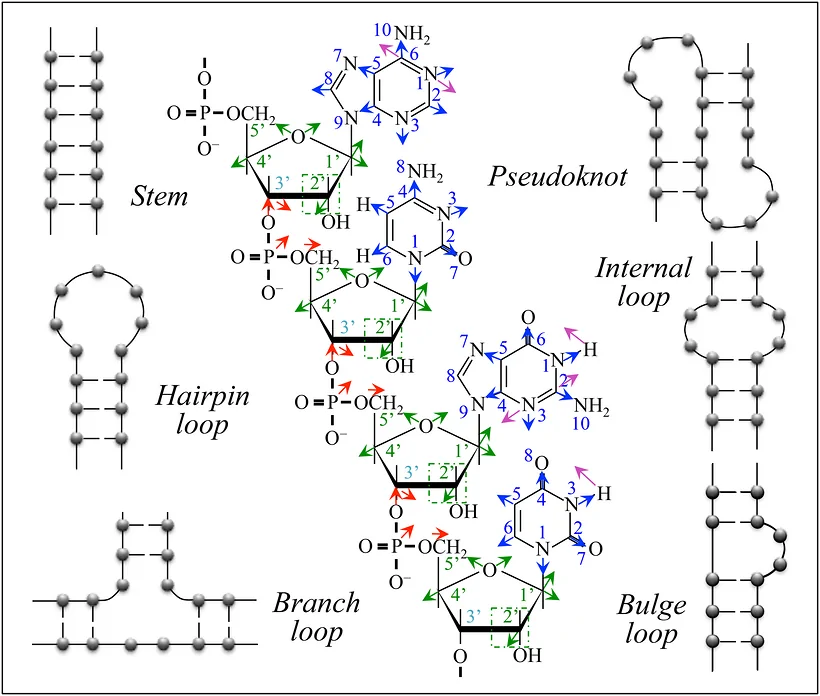

## 1 Introduction

The RNA of SARS‐CoV‐2 variants has been analyzed through a variety of methods aimed at understanding its genetic composition, mutations, and how those characteristics influence transmissibility, immune escape, and pathogenicity [1, 2, 3, 4, 5]. The primary tools so far employed in SARS‐CoV‐2 RNA analysis include: next‐generation sequencing (NGS) and reverse transcription polymerase chain reaction (RT‐PCR) [6, 7]. These analytical methods have allowed for precise identifications of the continuous mutations of viral RNA, especially with respect to genome regions responsible for the spike protein, which is critical for viral entry into human cells [8]. NGS, with its low error rates, nowadays represents the gold standard for sequencing viral RNA. NGS platforms are available, which have been used to successfully detect both single nucleotide polymorphisms and larger genetic shifts, such as deletions or insertions, which differentiated newly emerging SARS‐CoV‐2 subvariants [9]. However, since often requiring long reads for accurate genome assembly and for detecting complex genetic rearrangements, efforts in decoding the entire genome of a newly emerging virus by NGS might become quite time‐consuming and cumbersome. Mapping the complete SARS‐CoV‐2 viral genome has allowed researchers to track mutations over the entire RNA sequence [10], providing deep insights into the genetic evolution of SARS‐CoV‐2, including the identification of its new variants and subvariants (such as the omicron subvariants from BA.1, through BA.2 and BA.5, to XBB.1) [11, 12, 13]. Whole genome sequencing thus represents a crucial tool in understanding how such variants have emerged, how they differ from previous strains, and how their mutations have affected the overall virus ability to evade immunity or change its transmissibility. Note, however, that WGS, while extremely reliable in detecting single‐nucleotide variants, might eventually miss or misinterpret a variant [14]. Moreover, its intrinsically complex and multi‐step nature implies that standard applications of the WGS process might take weeks. RT‐PCR presently represents the most developed molecular technique for fast diagnose and detection of viruses [15]. It has covered a crucial role in SARS‐CoV‐2 early detection and evaluation of emerging variants, including surveillance and assessment of resistance profiling [16]. The PCR method provides results much more quickly as compared to WGS and NGS testing procedures, and could be designed to target multiple specific regions of virus genetic material, thus avoiding cross‐reactivity with other pathogens and ensuring accurate diagnosis [17]. Accordingly, PCR represents the most currently applied analytical platform employed from bench to bedside [18]. This method involves reverse transcription of the viral RNA into complementary DNA, which is then amplified. By using specific primers that target regions of the virus genome, RT‐PCR can confirm the presence of the virus and, in some cases, identify specific variants. Special assays have been developed to identify variants like Omicron (e.g., S‐gene target failure), based on specific mutations found in the spike protein gene [19]. On the limitation side, RT‐PCR requires the development of primers that are specific to the virus to be detected; and, if there are mutations or genetic variability in the viral genome, the primers may not work effectively, leading to missing detections [20]. In other words, RT‐PCR can only detect viruses for which the genetic sequence is already known. In the case of emerging variants or novel viruses, RT‐PCR assays might thus not be readily available, and developing them could take times as long as NGS.

Raman spectroscopy is a method of molecular diagnostics [21, 22], which has potential for revolutionizing both basic science and clinical practice of infectious diseases. Once fully developed, it could evolve from bench to bedside and provide yet missing molecular‐scale information. Raman spectroscopy is capable to unfold details that could either support or add new information to the presently available methods in virology. This could lead to a paradigmatic shift in acute‐care settings, where both timely and accurate diagnostics/classification of viral variants are critical for patient treatment decisions and outcomes. However, Raman spectroscopy cannot directly sequence or decode the genetic material of viruses. So far, it has been applied to rapid identifications of the viral presence of SARS‐CoV‐2 in patient samples, including nasal swabs, saliva, or blood, based on characteristic peaks that correlate with specific virus biochemical fingerprints or markers [23, 24]. It has also delivered precise information on the biochemical composition of SARS‐CoV‐2 as well as of other virus particles, which enabled to differentiate among variants and to discuss their degree of virulence [25, 26, 27, 28, 29]. In other words, Raman applications in virology have so far been more about viral detection and structural characterization, than on analyzing viral RNA sequences. In a previous paper [28], we showed how Raman analyses on whole virions could unfold differences in signals of SARS‐CoV‐2 subvariants, including those from RNA bases. In analogy with a previously proposed “genetic barcode”, which included information from a global database of genetic data extracted from coronavirus tests [30], we also proposed a “Raman barcode” capable to locate the presence of a multiplicity of slightly different versions of virus subtypes within their general classification into individual variants [25]. In particular, our previous studies showed that fractional data of Raman intensity did not fully correspond to base fractions obtained from genomic data, because they were altered by base pairing, signal cross‐sections, and the possible presence of viral nucleoproteins associated at preferential RNA sites [31]. Nevertheless, they represented a new set of “Raman genomic” data whose barcodes were univocally distinctive of virus variants and subvariants. This discovery suggested the possibility to define the new concept of “Raman genome”, as a tool encrypting insightful and promptly obtainable information in comparison with and complementarily to conventional genomic analyses.

In this paper, we further delve into the previously established concepts of Raman genome and Raman barcode by systematically collecting Raman spectra on RNA nucleotide strands extracted from 20 different SARS‐CoV‐2 variants and subvariants. Unlike the case of the above‐mentioned Raman studies on whole virions, the present spectroscopic characterizations directly capture and decrypt RNA structural differences at the molecular scale, without overlap/interference of other molecules present in the virions’ structure. Raman assessments succeeded in clearly differentiating SARS‐CoV‐2 viral strains, according to Raman signals characteristic of phosphodiester chain, ribofuranose rings, and nitrogenous bases only; signals from the two former ones being linked to nucleotide chain length and backbone conformation, while those from the latter ones being sensitive to RNA secondary structures. Such important spectral signatures, which defined the so‐called “Raman genome” for SARS‐CoV‐2 strains, were all encrypted into RNA Raman barcodes, whose systematization into a dedicated Raman library could, in the future, guide fast and precise virus classifications according to the spectroscopic response of their viral RNA molecular structure.

## 2 Experimental Procedures

### 2.1 Viral RNA Extraction

All SARS‐CoV‐2 variants used in this study were provided by the National Institute of Infectious Diseases (NIID), Japan. SARS‐CoV‐2 variants were first propagated in VeroE6/TMPRSS2 cells (JCRB1819: JCRB Cell Bank). Then, the respective viral RNAs were extracted from the culture supernatant using the NucleoSpin RNA Virus kit (TAKARA BIO, Otsu, Japan) according to the manufacturer's protocol.

### 2.2 Raman Spectroscopy and Machine Learning Algorithms

RNA Raman spectra were collected by means of a high spectrally resolved spectrometer designed for measurements on biological samples (LabRAM HR800, Horiba/Jobin‐Yvon, Kyoto, Japan). The equipment was set in confocal mode with a 20x objective lens and the wavelength of the incoming light was 532 nm, as generated by a solid‐state laser source operating at 10 mW. The Raman scattered light was monitored by means of a single monochromator interfaced with an air‐cooled charge‐coupled device (CCD) detector (Andor DV420‐OE322; 1024 × 256 pixels). The acquisition time for a single spectrum was typically 20 s for three successive acquisitions at each location. A spectral resolution better than 1 cm^−1^ could be achieved by concurrently collecting (during each measurement) an internal reference signal from a selected neon lamp to calibrate eventual scattering of the spectrometer. Series of 10 spectra were systematically collected at different locations (over a total area of ∼2 mm^2^) for each RNA sample and averaged in order to obtain statistically representative spectra. Data within selected spectral areas were also collected in the same way. Experimental Raman spectra were subjected to polynomial baseline subtraction and deconvolution into series of Gaussian‐Lorentzian sub‐band components. The baseline subtraction procedure was performed using options available in commercial software (LabSpec 4.02, Horiba/Jobin‐Yvon, Kyoto, Japan) with fixed criteria for all collected spectra. Spectral deconvolution criteria were based on machine learning algorithms, which utilized an automatic solver exploiting a linear polynomial expression of Lorentzian‐Gaussian functions [22]. Because, as seen in the remainder of this paper, Raman band intensities of nucleic acids are strongly influenced by the local RNA secondary structure through hyperchromic and hypochromic effects, the constrained optimization primarily exploits the reproducible spectral positions and full‐width‐at‐half‐maximum values of the elementary Raman bands, whereas their relative intensities were treated as structure‐dependent variables. Consequently, the optimization was performed globally over the entire spectrum rather than through a fixed sequence of local peak‐fitting operations. This computational procedure produced a case‐by‐case deconvolution with the closest match to the experimental spectrum, while additionally identifying the primary sub‐band contributing molecules (> 90%).

### 2.3 Principal Component Analysis and Data Statistical Validity

In the attempt to explore spectral variations among different RNA strands, principal component analysis (PCA) was applied [32]. Partial least‐squares regression (PLSR) was used to obtain calibration models between Raman spectra and the selected quality features [33]. Assessments of the number of components to be used in calibration models was substantiated through full cross‐validation and a test set was used for validation. The statistical relevance of the parameters extracted from Raman experiments was analyzed by computing mean values and standard deviations. Statistical validity was evaluated by applying the unpaired Student's *t*‐test. Values *p* < 10^−2^ were considered as statistically significant and labeled with two asterisks.

### 2.4 Prediction by Computational Assessment of RNA Secondary Structures

RNAfold analysis in the ViennaRNA ver. 2.7.0 package [34, 35] was conducted in order to obtain the predicted secondary structure based on RNA sequences of the 20 distinct SARS‐CoV‐2 lineages under examination. The nucleotide sequences were retrieved from the GISAID database (https://gisaid.org/) [36]. The GISAID accession numbers of these sequences are as follows:

EPI_ISL_408667, EPI_ISL_804007, EPI_ISL_1123289, EPI_ISL_877769, EPI_ISL_2158617, EPI_ISL_4204973, EPI_ISL_3869208, EPI_ISL_4470503, EPI_ISL_7418017, EPI_ISL_7571618, EPI_ISL_9595859, EPI_ISL_14328510, EPI_ISL_13969765, EPI_ISL_13278440, EPI_ISL_15740139, EPI_ISL_13241867, EPI_ISL_13241868, EPI_ISL_15579783, EPI_ISL_15669344, and EPI_ISL_12703378.

Using the predicted secondary structure, we calculated four descriptors at each nucleotide position by means of a custom Python script that parsed the RNAfold partition‐function output, the base‐pair probability dot‐plot file. The base‐pairing probability, BP(*i*), is the probability that nucleotide *i* is base‐paired with any partner in the thermodynamic ensemble of structures, and was obtained by summing the McCaskill base‐pair probabilities P(*i*,*j*) over all partners *j* [the “ubox” entries of the dot‐plot, which store √P(*i*,*j*)]. BP(*i*) ranges from 0 for positions that are consistently single‐stranded and accessible to 1 for positions that are paired in essentially all structures of the ensemble. The cumulative minimum free energy (MFE) structure profile, cMFE(*i*), is a mountain‐plot representation of the MFE structure (the “lbox” entries), in which each MFE base pair (*i*,*j*) increments every position it encloses by one, so that cMFE(*i*) equals the number of MFE base pairs enclosing nucleotide *i*, which corresponds to its nesting depth. cMFE is therefore topological rather than energetic in nature, with larger values marking nucleotides embedded within deeply nested helices, and it is taken as an index of the local structural content of the MFE structure. The Shannon entropy, SE(*i*), is the positional entropy of the pairing distribution, SE(*i*) = −Σ_j_ P(*i*,*j*) ln P(*i*,*j*) − q(*i*) ln q(*i*), where q(*i*) = 1 − Σ_j_ P(*i*,*j*) is the probability that *i* is unpaired (values in nats); it is close to zero where a nucleotide adopts an almost unique pairing state and large where several alternative pairings coexist, and thus quantifies the structural uncertainty at each position. The strain score, SS(*i*), is a heuristic composite descriptor defined as SS(*i*) = BP(*i*) × cMFE(*i*) × SE(*i*), so that it becomes large only where a nucleotide is simultaneously likely to be paired, deeply embedded in nested structure, and conformationally uncertain, corresponding to positions that are structurally engaged yet unstable and that we can interpret as being under local structural strain. SS is not a standard thermodynamic quantity but a custom index introduced here to flag such labile but structured regions. For each descriptor, both the position‐wise values and their running cumulative sums along the genome were computed.

In addition, relplot.pl in the ViennaRNA package was used to generate a figure of the predicted secondary structure. We also calculated the total number of the following structural elements from the dot‐bracket sequences using a custom Python script: stem, hairpin loop, bulge loop, internal loop, and multibranched loop. In this context, a hairpin loop was defined as a loop region enclosed by a base pair and containing at least three unpaired bases.

## 3 Experimental Results

### 3.1 Average Raman Spectra of SARS‐CoV‐2 RNA Nucleotide Strands

Figure 1 shows average Raman spectra in the wavenumber interval 400–1800 cm^−1^ as recorded for the RNA structures of 20 different SARS‐CoV‐2 variants/subvariants (cf. labels in inset corresponding to assignments in Table 1). Each of the presented spectra is an average of 10 collected at different locations on the same RNA sample. All spectra in Figure 1 displayed morphological similarity, but they clearly differed in a number of fundamental details. Proposed vibrational origins for the main bands detected in the wavenumber interval 400–1800 cm^−1^ are given in Table 2 with related references. According to published literature [37], the most prominent Raman markers for different bases are expected to relate with base ring structures in the low‐wavenumber zone 650–850 cm^−1^: at ∼724 cm^−1^ (ring breathing in adenine), ∼778 cm^−1^ (ring breathing in cytosine), ∼672 cm^−1^ (6‐ring breathing in guanine), and ∼791 cm^−1^ (ring breathing in uracil). The vibrational origins of purines/pyrimidines Raman markers are schematically depicted in Figure 2a together with spectra collected on the four elementary RNA bases (replotted in (Figure 2b–e, Ref. [37]). Pyrimidine signals in virus RNA showed relative Raman intensities systematically higher than the correspondent ones in purines for all variants/subvariants (cf. labels in inset to Figure 1). Such scatters cannot be explained by invoking differences in base fractions, since base fractions assessed by genomic analyses did not differ to such an extent (cf. Table 1). The origin of different relative intensities for both purine and pyrimidine ring‐breathing signals mainly resides in the different cross sections of the above Raman signals and, partly, from the presence of hairpin loops and stem regions in the RNA strands [38]. In a hairpin loop, the relative intensity of Raman signals from the stem (double‐stranded) and the loop (single‐stranded) differs, the stem typically having sharper and more intense features related to base pairing and stacking, while the loop displaying broader and weaker signals due to the unpaired nature of the bases. In other words, Raman bands arising from ring vibrations of specific purine bases might either increase or decrease in intensity as a function of the presence of specific secondary structures, a phenomenon generally referred to as Raman hyper and hypochromism, respectively [39]. In other words, the Raman intensities discussed herein cannot be simply interpreted as being directly proportional to the genomic abundance of individual nucleotides, since the effective Raman scattering of nucleobases embedded within intact RNA is strongly modulated by their local structural environment, including base stacking, hydrogen bonding, and backbone conformation. It should be noted at the outset that the occurrence of bold spectral differences compared to relatively small genomic differences could be seen as a path toward easier/faster determination of variants/subvariants. This point will be discussed in more details in a later section.

**FIGURE 1:**
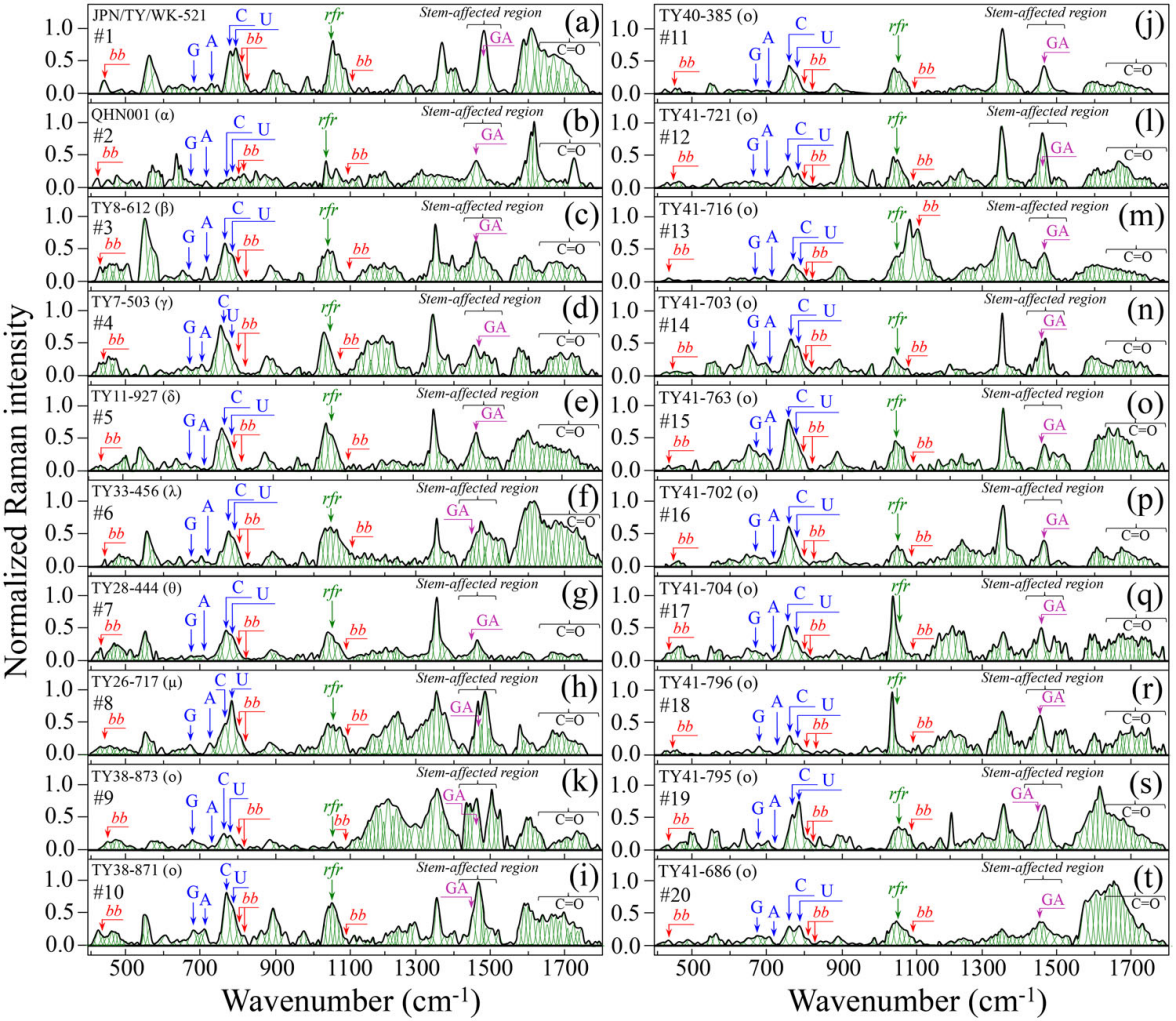
In (a–t), Raman spectra of RNA strands from 20 different SARS‐CoV‐2 variants/subvariants in the wavenumber interval 400–1800 cm^−1^ (labels in inset are the same as those in Table 1); each spectrum is the average of 10 spectra collected at different locations on the same RNA sample. Abbreviations are, as follows: phosphodiester backbone (*bb*), guanine (G), Adenine (A), cytosine (C), uracil (U), and ribofuranose ring (*rfr*).

**TABLE 1:** List of the investigated SARS‐CoV‐2 variants/subvariants with their total base contents and respective base fractions as computed by means of the ViennaRNA software package; apparent base fractions computed from the fractional areas of base ring signals (and affected by Raman cross‐section effects) are also shown for comparison.

Label	Variant	G (Raman)	G (genome)	A (Raman)	A (genome)	C (Raman)	C (genome)	U (Raman)	U (genome)	Total base content
#1	JPN/TY/WK‐521	10.0%	19.6%	5.6%	29.9%	40.6%	18.4%	43.8%	32.1%	29878 (+0.44%)
#2	QHN001 (α)	20.0%	29.9%	21.9%	32.1%	25.0%	19.6%	33.1%	18.4%	29719 (−0.10%)
#3	TY8‐612 (β)	6.4%	19.6%	11.6%	29.9%	48.6%	18.3%	33.4%	32.2%	29789 (+0.14%)
#4	TY7‐503 (γ)	8.4%	19.6%	9.0%	29.9%	49.5%	18.4%	33.1%	32.1%	29777 (+0.10%)
#5	TY11‐927 (δ)	10.7%	19.6%	2.6%	29.9%	48.6%	18.3%	38.1%	32.2%	29769 (+0.07%)
#6	TY33‐456 (λ)	10.4%	19.6%	5.9%	29.9%	53.1%	18.3%	30.6%	32.2%	29752 (+0.01%)
#7	TY28‐444 (θ)	6.1%	19.6%	3.8%	29.9%	49.9%	18.3%	40.1%	32.2%	29763 (+0.05%)
#8	TY26‐717 (µ)	11.8%	19.6%	11.6%	29.9%	31.9%	18.3%	44.7%	32.2%	29781 (+0.11%)
#9	TY38‐873 (ο)	9.5%	19.6%	12.2%	29.9%	37.3%	18.3%	41.0%	32.2%	29752 (+0.01%)
#10	TY38‐871 (ο)	14.9%	19.6%	12.3%	29.9%	43.3%	18.3%	29.5%	32.2%	29752 (+0.01%)
#11	TY40‐385 (ο)	5.4%	19.6%	4.3%	29.9%	50.0%	18.3%	40.3%	32.2%	29729 (−0.07%)
#12	TY41‐721 (ο)	16.1%	19.6%	8.7%	29.9%	51.2%	18.3%	24.0%	32.2%	29729 (−0.07%)
#13	TY41‐716 (ο)	12.2%	19.6%	6.5%	29.9%	52.0%	18.3%	29.3%	32.2%	29729 (−0.07%)
#14	TY41‐703 (ο)	14.8%	19.6%	3.9%	29.9%	44.5%	18.3%	36.8%	32.2%	29714 (−0.12%)
#15	TY41‐763 (ο)	16.6%	19.6%	6.4%	29.9%	44.4%	18.3%	32.6%	32.2%	29714 (−0.12%)
#16	TY41‐702 (ο)	15.9%	19.6%	4.0%	29.9%	53.0%	18.3%	27.1%	32.2%	29723 (−0.09%)
#17	TY41‐704 (ο)	12.9%	19.6%	7.4%	29.9%	51.8%	18.3%	27.1%	32.2%	29723 (−0.09%)
#18	TY41‐796 (ο)	20.7%	19.6%	3.6%	29.9%	42.5%	18.3%	33.2%	32.2%	29723 (−0.09%)
#19	TY41‐795 (ο)	6.6%	19.6%	7.1%	29.9%	40.4%	18.3%	45.9%	32.2%	29726 (−0.08%)
#20	TY41‐686 (ο)	16.8%	19.6%	2.0%	29.9%	37.5%	18.3%	43.7%	32.2%	29726 (−0.08%)

**TABLE 2:** Proposed vibrational origins for the main bands detected in the wavenumber interval 400–1800 cm^−1^ are given in Table 2 with related references.

Label	Wavenumber (cm^−1^)	Vibrational origin	Refs.
1	**432**	**C3’−O−P bending in RNA backbone**	[39]
2	468	C−C−O bending in ribofuranosyl ring	[44]
3	547	O−C−O bending in ribofuranosyl ring	[44]
4	606	in‐plane ring deformation in uracil	[38]
5	626	ribose ring deformation	[44]
6	640	in‐plane ring deformation in uracil	[38]
7	653	out‐of‐plane 5‐ring deformation in guanine	[38]
8	**672**	**6‐ring breathing in guanine**	[38]
9	691	5‐ring out‐of‐plane deformation in adenine	[38]
10	703	N2−C2−N1 in‐plane ring deformation in guanine	[38]
11	**724**	**ring breathing in adenine**	[38]
12	739	C−O−C bending in ribose ring	[44]
13	757	C5−C4−N9 in‐plane ring deformation in guanine	[38]
14	**778**	**ring breathing in cytosine**	[38]
15	**791**	**ring breathing in uracil**	[38]
16	**813**	**O−P−O stretching in RNA backbone**	[39]
17	**824**	**O−P−O antisymmetric stretching in RNA backbone**	[39]
18	840	C−C symmetric stretching in ribose ring	[44]
19	854	N1−C2−N3 & C4−N9−C8 in‐plane ring deformation in guanine	[38]
20	867	C8−H & N7−H out‐of‐plane deformation in guanine	[38]
21	889	C−C stretching & C−CH in‐plane bending in ribofuranosyl ring	[38]
22	906	C−O stretching in ribose ring	[44]
23	926	N7−C8−N9 in‐plane ring deformation in guanine 6‐ring in‐plane deformation in adenine	[38]
24	945	5‐ring in‐plane deformation in guanine	[38]
25	1015	C−O stretching in ribofuranosyl ring	[44]
26	1026	NH_2_ rocking in adenine C−C stretching in ribofuranosyl ring	[38] [44]
27	1045	**C−C & C−O symmetric stretching in ribofuranosyl rings**	[44]
28	1063	C−C stretching in ribofuranosyl ring N1−C2 stretching in guanine ring	[44] [38]
29	1094	PO_2_^−^ symmetric stretching	[41]
30	1144	NH_2_ rocking in guanine C−N ring stretching in guanine C8−N9 stretching in adenine in plane N9−H bending in adenine	[38]
31	1233	N5−N7 and N7−C8 stretching in guanine	[38]
32	1250	in‐plane ring deformation in cytosine, uracil and adenine	[38] [46]
33	1273	in plane C8−H and N9−H bending in adenine N7−C8 stretching in adenine	[38]
34	1299	ring deformation in cytosine and adenine C−N ring stretching in guanine in plane C8−H, N1−H and N10−H bending in guanine	[46] [38] [38]
35	1320	ring deformation in guanine	[46]
36	1338	ring deformation in adenine	[46]
37	1368	ring deformation in guanine and adenine	[46]
38	1390	CH_2_ wagging	[47]
39	1413	ring deformation in adenine, guanine and cytosine in plane N1−H, C6−H and C5−H bending in uracil	[46] [38]
40	**1460**	**in plane C=N ring stretching in guanine in plane C8−H, N1−H and N10−H bending in guanine. in plane C2=N3, N1=C6 stretching in adenine in plane C2−H bending in adenine NH_2_ scissoring in adenine**	[47] [38] [38] [38] [38] [38]
41	1483	ring deformation in guanine and adenine N7−C8 stretching in adenine in plane C8−H bending in adenine NH_2_ scissoring in adenine	[46] [38] [38] [38]
42	1533	in plane C5−C6 and C6−N1, stretching in uracil in plane C6−H bending in uracil	[38]
43	1573	ring deformation in adenine and guanine	[46]
44	1595	C4=O, C5−C6 and C2=O stretching in uracil in plane N1−H and C6−H bending in uracil	[38]
45	1654	C4=O and C2=O stretching in uracil	[38]
46	1664	C6=O and C5−C6 stretching in guanine. in plane N1−H bending in guanine NH_2_ scissoring in guanine	[38]
47	1692	C=O stretching in uracil	[46]
48	1720	C=O stretching in guanine	[46]

**FIGURE 2:**
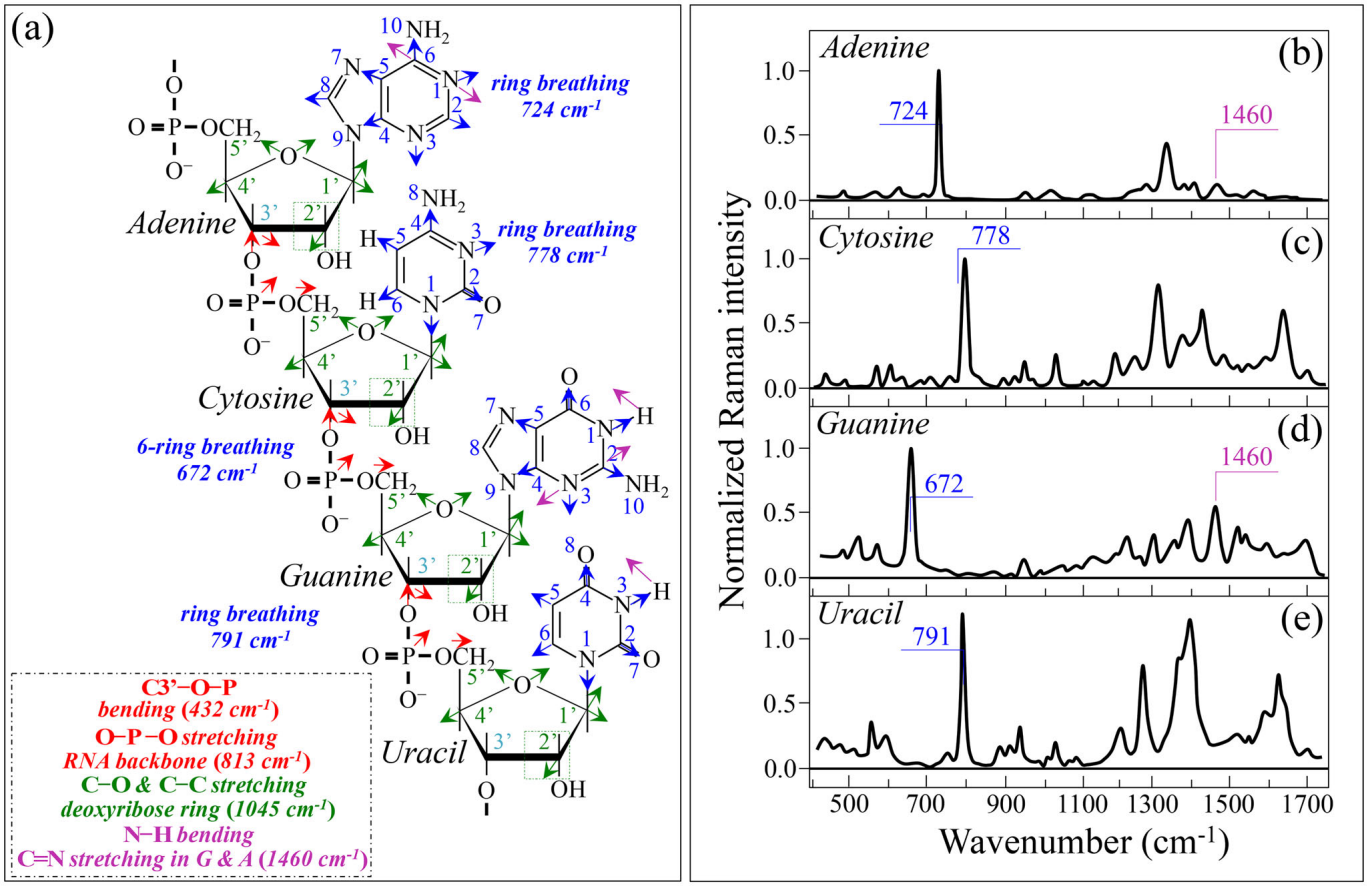
(a) Schematic draft of the RNA strand and vibrational origins of the selected Raman markers for purines and pyrimidines, for the phosphodiester backbone, and for the ribofuranose ring. Raman reference spectra of the four elementary RNA bases are given in (b–e) (replotted from Ref. [37]; labels in inset are in wavenumber and the abbreviations are the same as those given in Figure 1).

Recorded as a high‐wavenumber shoulder to the uracil ring‐breathing band, the O−P−O stretching band of phosphodiester backbone appears at ∼807 cm^−1^, with its antisymmetric pair at ∼824 cm^−1^ (cf. labels in inset to Figure 1 and Table 2) [40]. Note that also phosphate‐related bands could exhibit signatures that are sensitive to the specific secondary structure of RNA. In the presence of hairpins, the loop causes the backbone to adopt different conformations compared to the stem, which in turn alter signal intensity [38, 41]. The 814 cm^−1^ band could be absent (or very broad) and with a shifted frequency in highly disordered (separated) chains, while it becomes quite pronounced in the presence of specific helical complexes. This band was reported to represent a strong indicator for the helical content of poly‐adenine/uracil complexes [42]. Further discussions on phosphodiester backbone bands will be given later.

An additional low‐wavenumber backbone signal of interest in this study is the one related to C3’−O−P bending at ∼432 cm^−1^ (cf. Figure 2 and Table 2) [31]. RNA spectra in Figure 1 include a further zone of interest at higher wavenumbers, which contains spectral contributions from β‐D‐ribofuranose rings at 1020–1060 cm^−1^. This zone is comprehensive of a relatively strong signal from C−O/C−C stretching at ∼1045 cm^−1^ (cf. Figures 1 and 2a) [42, 43]. The relative intensity of this ribofuranose ring signal was variable (cf. labels in Figure 1), being likely the most straightforwardly affected by genome length (cf. Table 1). This point will be further discussed in the forthcoming Section 4.1. Two neighboring bands at around 1026 and 1063 cm^−1^ include composite contributions from ribofuranose rings (cf. Table 2). An additional band related to PO_2_^−^ symmetric stretching in the RNA backbone was reported at ∼1094 cm^−1^ (cf. Table 2) [38]. However, this band was generally quite weak in all the present RNA samples and strongly fluctuated in its wavenumber position, thus making its detection difficult and its characteristics of difficult interpretation. Between approximately 1100 and 1300 cm^−1^, ring‐related stretching signals strongly overlap with in plane C−H and N−H bending (cf. Table 2) showing significant intensity variations among different RNA samples (cf. Figure 1). Relatively strong signals could also be detected within the interval 1300–1400 cm^−1^ (additional ring deformation modes overlapping C−H and N−H bending, wagging and scissoring modes, as shown in Table 2; cf. Figure 1) [37], at 1450–1490 cm^−1^ (C5’H_2_ scissoring overlapping with additional ring modes in purines) [44], at 1600–1700 cm^−1^ (ring modes and shifted carbonyl stretching in hairpin loops) [45], and 1700–1750 cm^−1^ (carbonyl stretching from uracil, cytosine and guanine) [38] (cf. Table 2 and labels in inset to Figure 1). Note, however, that differences in spectral intensities in these latter regions are of difficult interpretation. For example, two adenine‐related signals at 1299 and 1483 cm^−1^ (cf. Table 2) are expected to undergo intensity variations upon stem formation. Nevertheless, neither of these two bands can be used as a good indicator of adenine stacking since the effect of the overlapping signals, which lack hypo/hyperchromic dependence, significantly reduce their fluctuations related to stacking [42]. In other words, the majority of Raman signals in the spectral region between ∼1350 and 1600 cm^−1^, which includes both ring breathing and deformation modes, despite undergoing clear fluctuations in relative Raman intensity, can hardly be linked to the secondary structure of RNA. In published literature, the Raman bands measured at ∼1368, 1483, and 1573 cm^−1^ are unanimously assigned to adenine and guanine residues only [31, 39, 41, 42, 45, 46, 47]. The first two of these bands have been reported to be hyperchromic by some authors [46, 48], whereas other authors suggested that the last two bands are hypochromic [39, 46]. Data presented by Hobro et al. [38] solved this conundrum by showing that all these bands are indeed hyperchromic, the introduction of pyrimidine bulge likely reducing the presence of face‐to‐face RNA base stacking and thus limiting any related decrease in band intensity. Another prominent signal in this zone is found at around 1460 cm^−1^; this band represents a complex combination of ring signals and C−H and N−H bending/scissoring in purines only (cf. Table 2). All these signal components should contribute to an increased intensity (hyperchromism) of this band in the case of double‐stranded RNA, due to the higher stability arising from base pairing [45, 49]. In other words, the signal at 1460 cm^−1^ is expected to be sharper and more intense in RNA structures with a higher number of hairpins (and related stem length). Based on the above reasoning, the spectral region at around 1460 cm^−1^ was generally labeled as “stem‐affected region” in Figure 1.

An additional region potentially affected by the RNA secondary structure is the one at around 1590–1750 cm^−1^, which includes carbonyl stretching vibrations in purines and could change markedly upon hydrogen bond formation in the helix (cf. labels in inset to Figure 1 and Table 2). As a matter of fact, the relative intensities in the hairpin‐affected zone and the C═O zone appear to be somewhat related (cf. Figure 1). However, spectral shifts related to a complex architecture of intra‐base hydrogen bonds and the significant overlap of these signals between guanine and uracil render it very complex an interpretation of this spectral zone in order to implement a criterion of variants’ differentiation.

Figures 3, 4, 5, 6 show high spectrally resolved, averaged, and deconvoluted Raman spectra in the wavenumber regions 400–500, 600–950, 1000–1100, and 1400–1550 cm^−1^, respectively, for the 20 SARS‐CoV‐2 variants/subvariants under investigation (cf. labels in inset). Selected bands from these deconvoluted high‐resolution regions are emphasized in different colors and will be discussed in detail in the forthcoming Sections 4.1–4.4.

**FIGURE 3:**
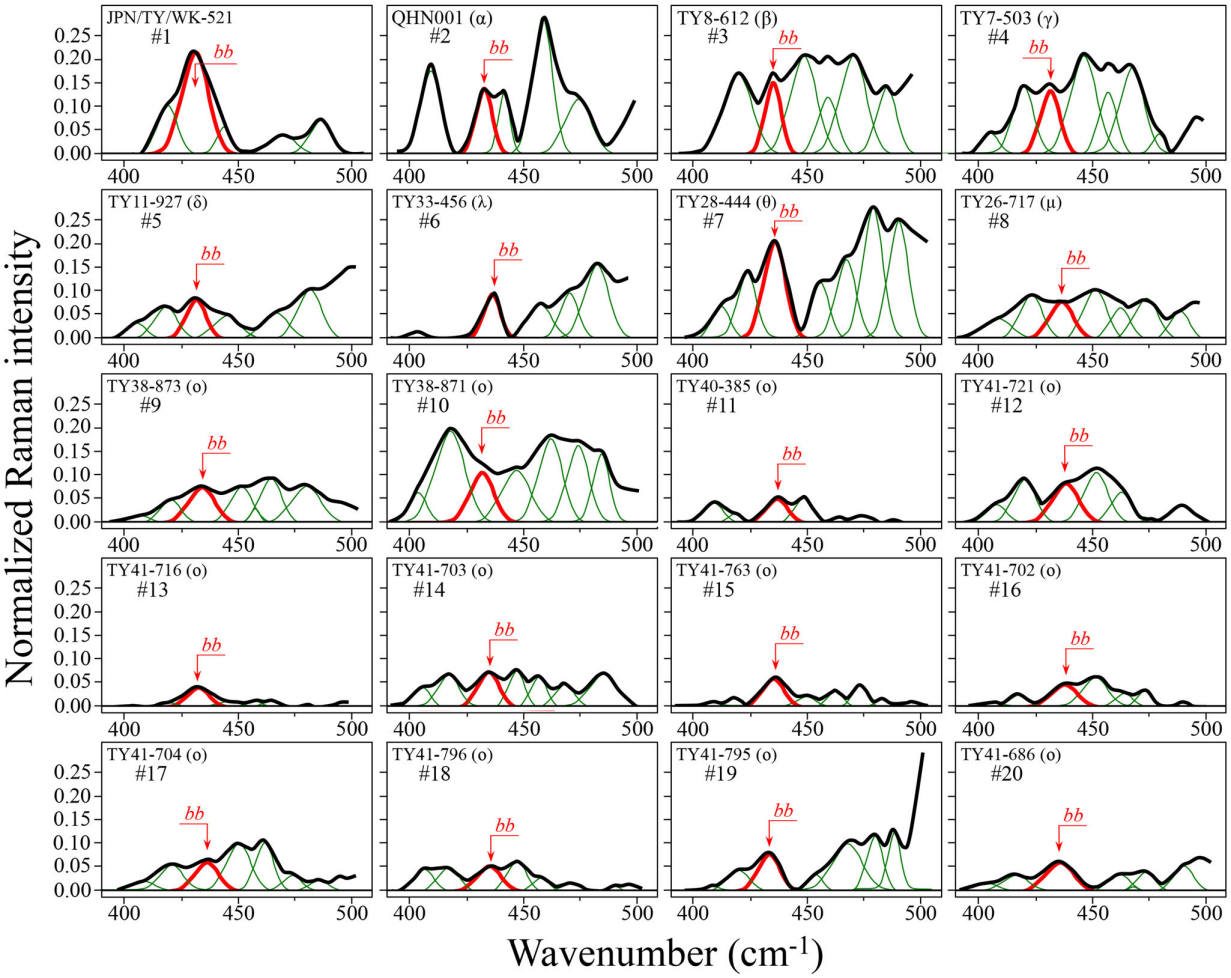
High spectrally resolved, averaged, and deconvoluted Raman spectra in the wavenumber region 400–500 cm^−1^ for the 20 SARS‐CoV‐2 variants/subvariants under investigation (cf. labels in inset). The deconvoluted *bb* band at ∼432 cm^−1^ (emphasized in red) will be discussed in detail in the forthcoming Sections 4.1–4.4.

**FIGURE 4:**
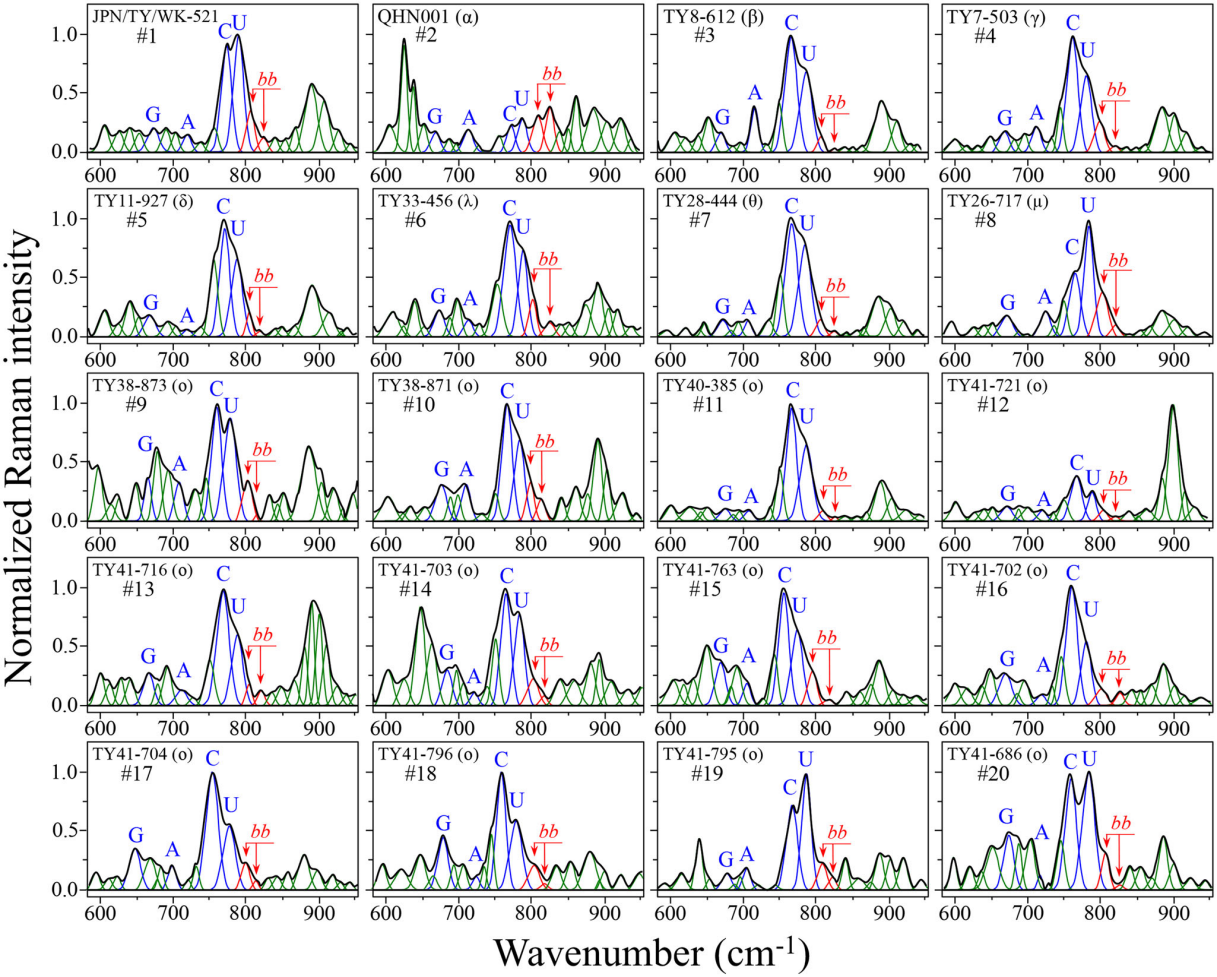
High spectrally resolved, averaged, and deconvoluted Raman spectra in the wavenumber region 600–950 cm^−1^ for the 20 SARS‐CoV‐2 variants/subvariants under investigation (cf. labels in inset). Selected marker bands (emphasized in blue and red color) in this region will be discussed in detail in the forthcoming Sections 4.1–4.3.

**FIGURE 5:**
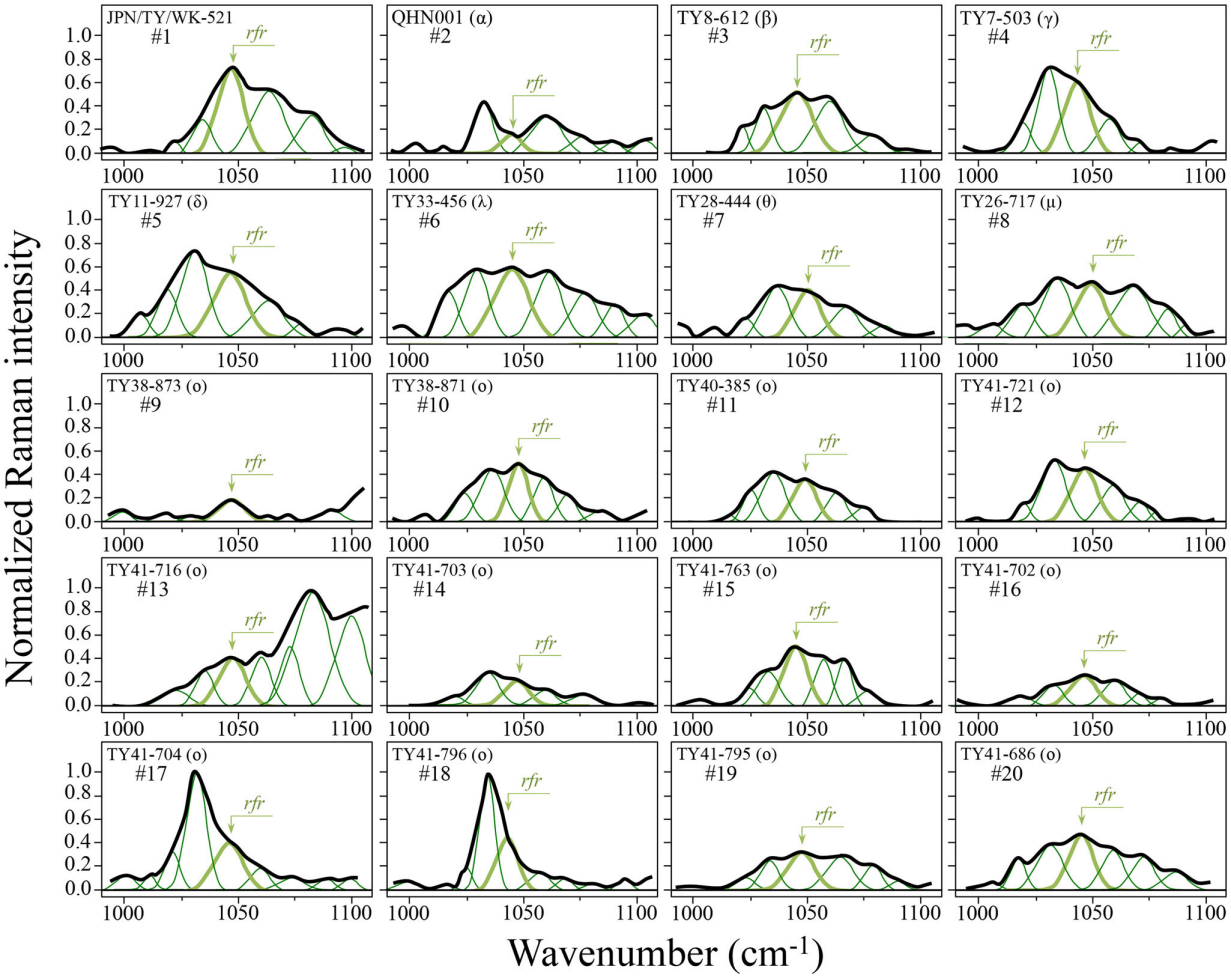
High spectrally resolved, averaged, and deconvoluted Raman spectra in the wavenumber region 1000–1100 cm^−1^ for the 20 SARS‐CoV‐2 variants/subvariants under investigation (cf. labels in inset). The deconvoluted *rfr* band at ∼1045 cm^−1^ (emphasized in light green) will be discussed in detail in the forthcoming Sections 4.1–4.4.

**FIGURE 6:**
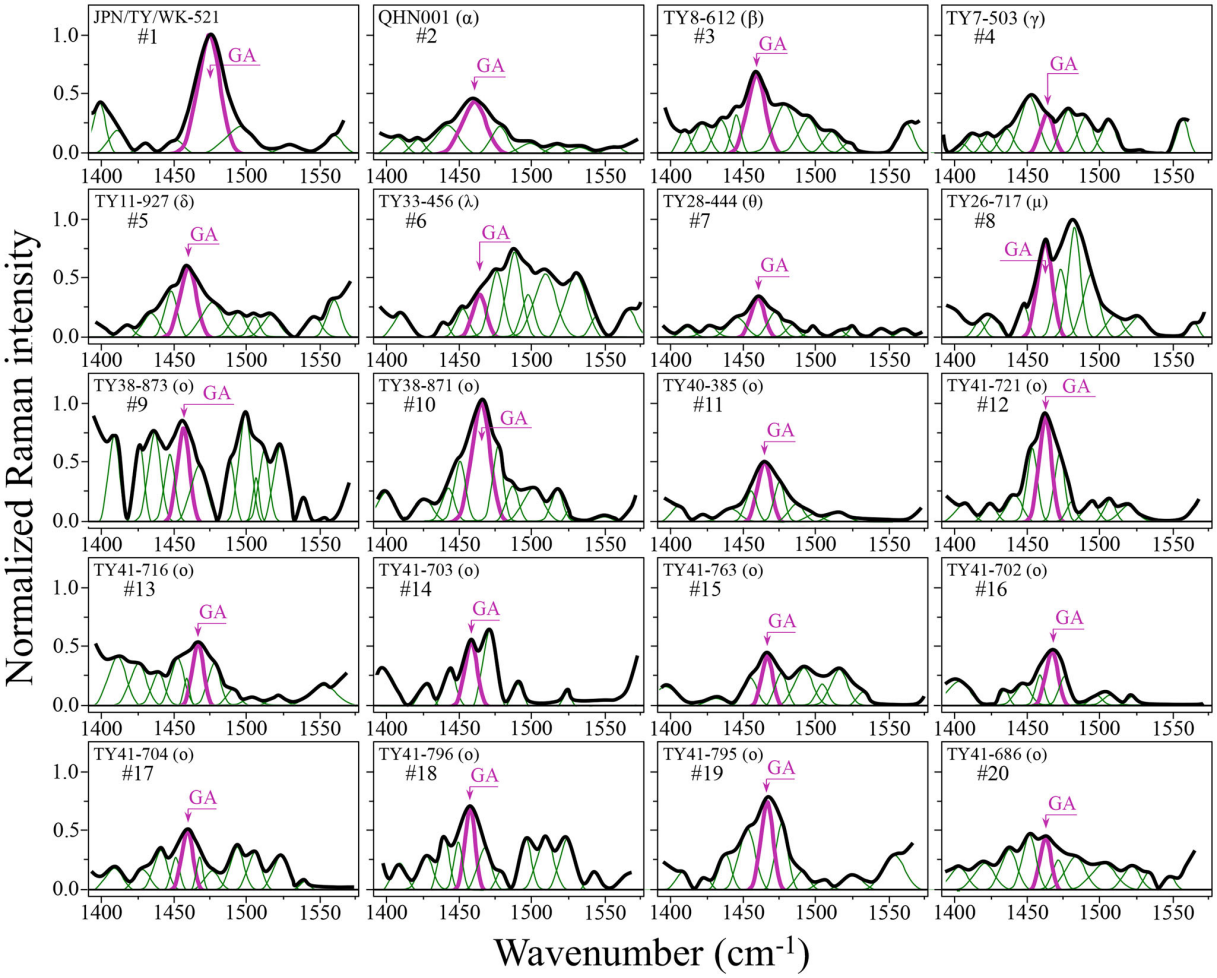
High spectrally resolved, averaged, and deconvoluted Raman spectra in the wavenumber region 1400–1550 cm^−1^ for the 20 SARS‐CoV‐2 variants/subvariants under investigation (cf. labels in inset). The deconvoluted GA band at ∼1045 cm^−1^ (emphasized in violet color) will be discussed in detail in the forthcoming Sections 4.1–4.4.

### 3.2 Principal Component Analysis and Raman Barcode Classification

As shown in the previous section, the Raman spectra collected on the RNA series of 20 SARS‐CoV‐2 variants/subvariants under investigation, despite displaying clear similarities, also showed distinctly different features. Accordingly, we first attempted to refine vibrational analyses through PCA as a procedure to enable univocal determination of RNA strands belonging to different variants/subvariants. Figure 7a–c display plots for PC3/PC1, PC3/PC2, and PC2/PC1 loading vectors, respectively, with reference to the entire Raman spectral region 300–1800 cm^−1^. As seen, PCA allows distinguishing among different samples only in the case of a limited number of variants/subvariants. In other words, we came across a partial failure in the identification of a large majority of SARS‐CoV‐2 RNA strands for any of the three different combinations of PCA loading vectors. The PCA analyses in Figure 7 stigmatize the extreme complexity of RNA secondary structure and call for a different approach to rationalize Raman analyses.

**FIGURE 7:**
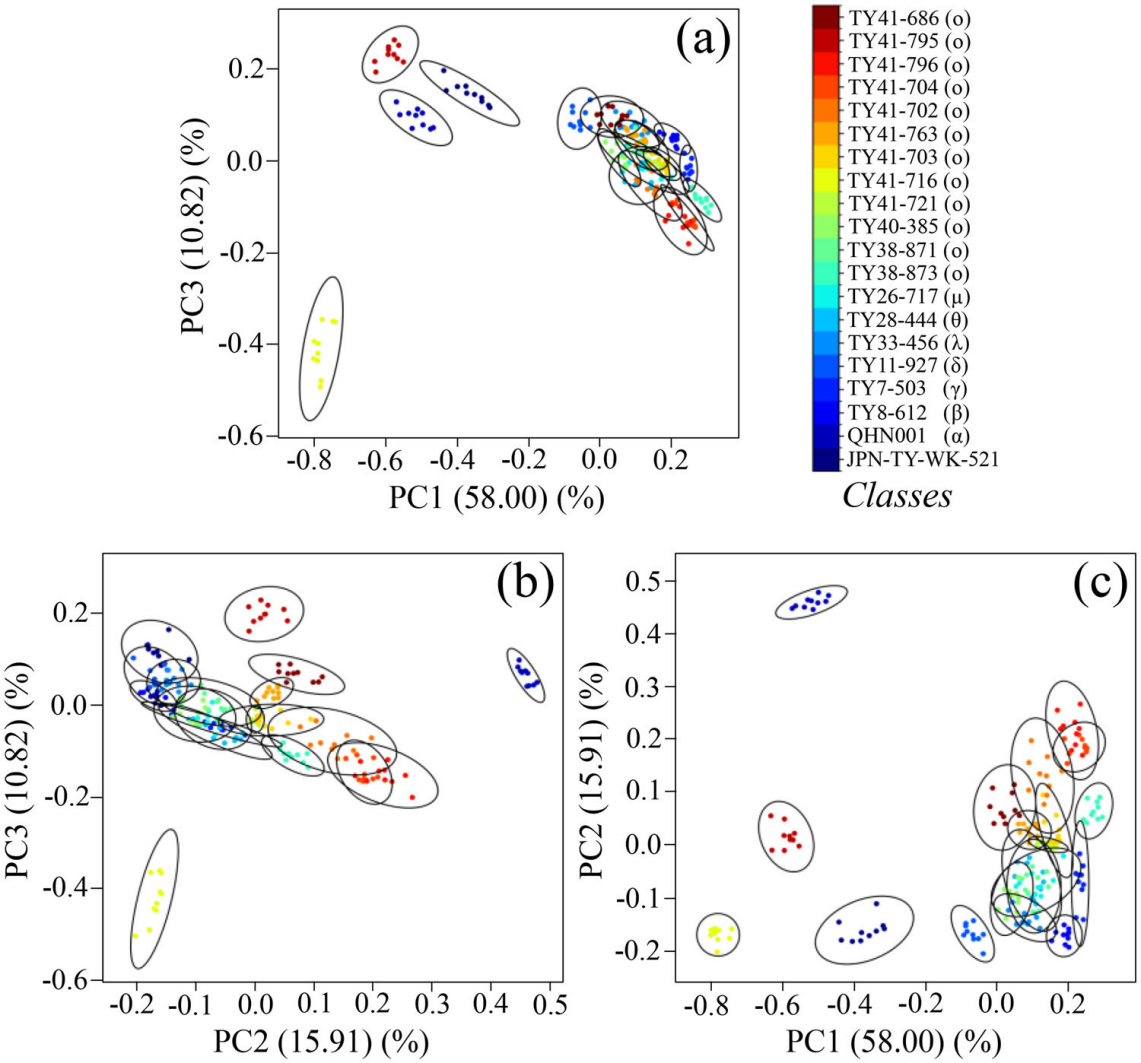
Principal component plots for (a) PC3/PC1, (b) PC3/PC2, and (c) PC2/PC1 loading vectors with reference to the entire Raman spectral region 300–1800 cm^−1^. As seen, PCA allowed distinguishing among different samples only for a limited number of variants/subvariants.

Figure 8a shows a draft for a proposed way to generate a barcode from a deconvoluted Raman spectrum [25, 50, 51]. In Figure 8b, the sub‐band sequence of the full Raman RNA spectra is converted into barcodes according to the algorithm in (a) (i.e., upon assigning to each band a line with thickness equal to 1/50 of the sub‐band width at half maximum and a distance from the successive line proportional to the band area). Each line in the barcodes corresponds to a specific peak in the Raman spectrum (cf. labels in inset). The barcodes built up with the above criterion univocally located the peculiar molecular structures of any of the investigated RNA samples including the spectroscopic characteristics deriving from their peculiar secondary structures.

**FIGURE 8:**
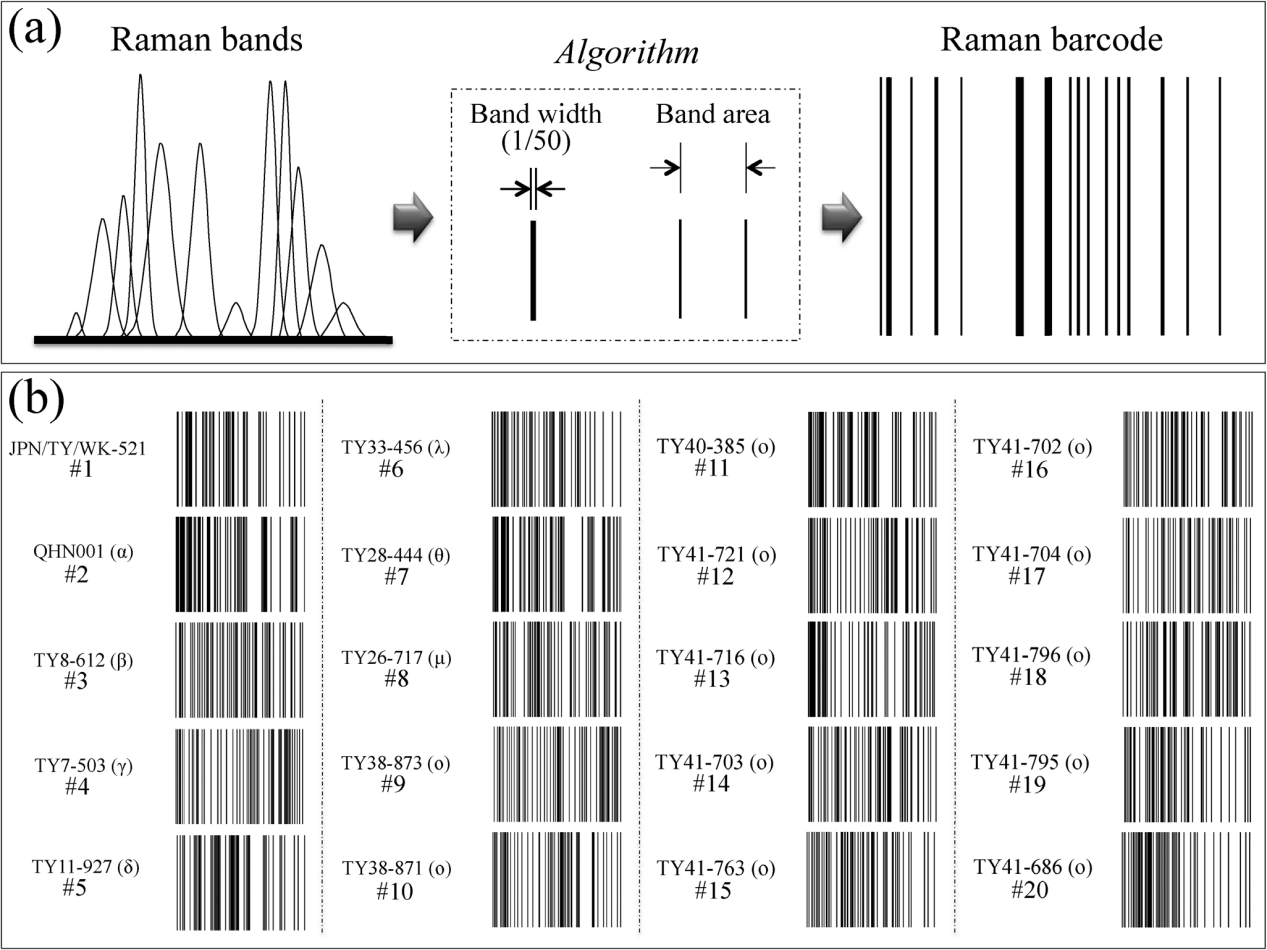
(a) Draft for a proposed way to generate a barcode from a deconvoluted Raman spectrum;^25,50,51^ in (b), the sub‐band sequence of the full Raman RNA spectra is converted into barcodes according to the algorithm in (a). All barcodes built up with the above criterion were different to each other, thus univocally locating the peculiar molecular structures of the investigated RNA samples.

### 3.3 Computational Results for RNA Secondary Structures

RNA secondary structure basically comprises stems, hairpin loops, pseudoknots, bulge loops, internal loops, and branch loops (cf. Figure 9a–f, respectively). In this study, the secondary structures of 20 SARS‐CoV‐2 linages were predicted using their RNA sequences via the ViennaRNA package. From the computed structures, we calculated the fractions of dimers and trimers in the genome sequence (Figures S1 and S2, respectively). In addition, we calculated 4 RNA secondary structural metrics (cMFE, BP, SE, and Strain Score; cf. Section 2.4). Figure 10 shows a structural map including genome strain score Figure 10a, the results of the calculation of the above parameters Figure 10b, and their cumulative values Figure 10c as a function of nucleotide position for the strain JPN/TY/WK‐521 (#1 Sample). Figure 11 summarizes the computed RNA secondary structures for all the studied strains (cf. labels in inset). Table 3 lists the salient parameters used in the following discussion to explore correlations between Raman signatures and the structural elements and to build up algorithms that link secondary structures to selected Raman spectroscopic features.

**FIGURE 9:**
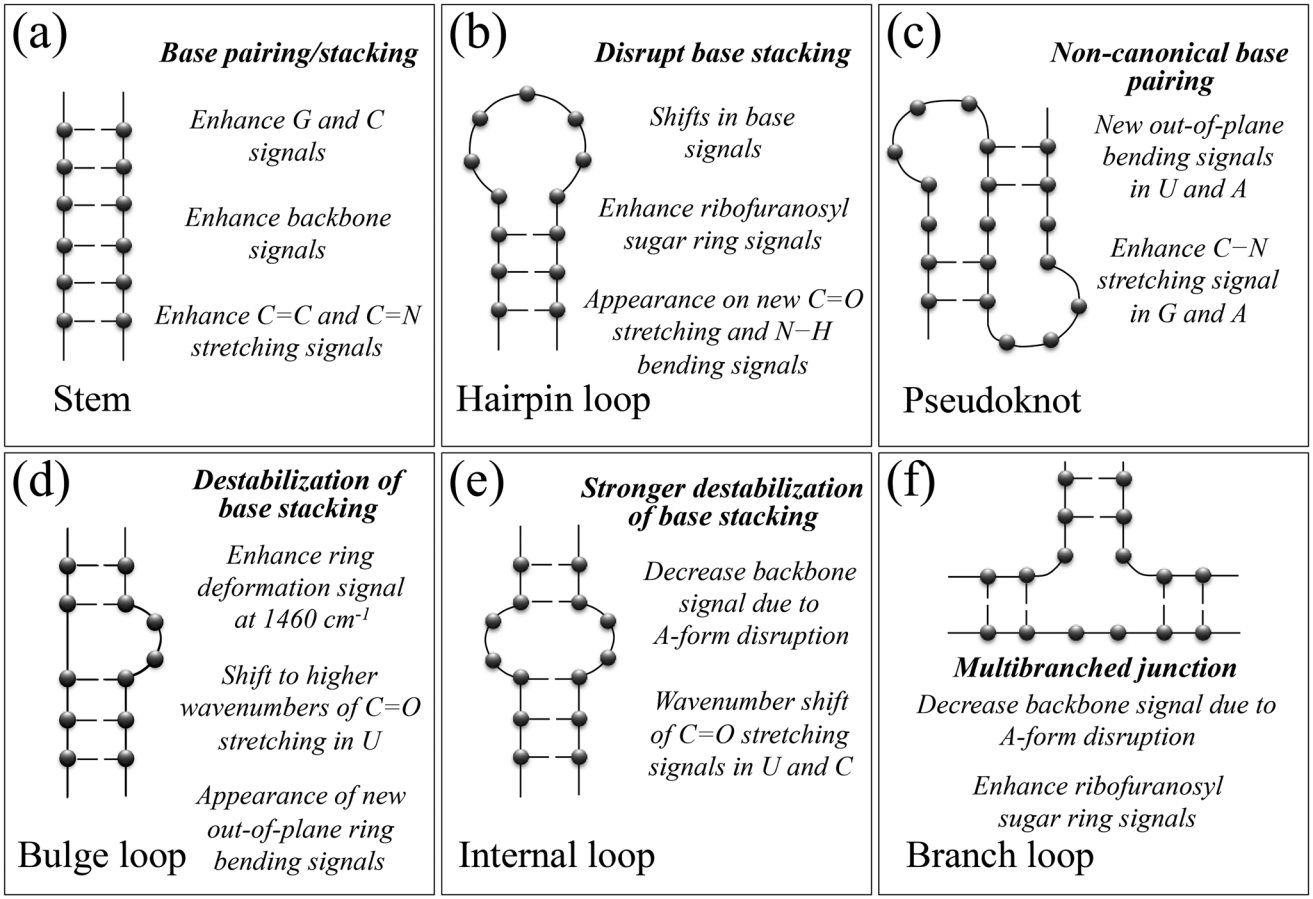
Schematic drafts of RNA secondary structures: (a) stem, (b) hairpin loop, (c) pseudoknot, (d) bulge loop, (e) internal loop, and (f) branch loop; in inset, list of Raman signals (and related mechanisms) that are expected to be affected by the presence of the respective structures.

**FIGURE 10:**
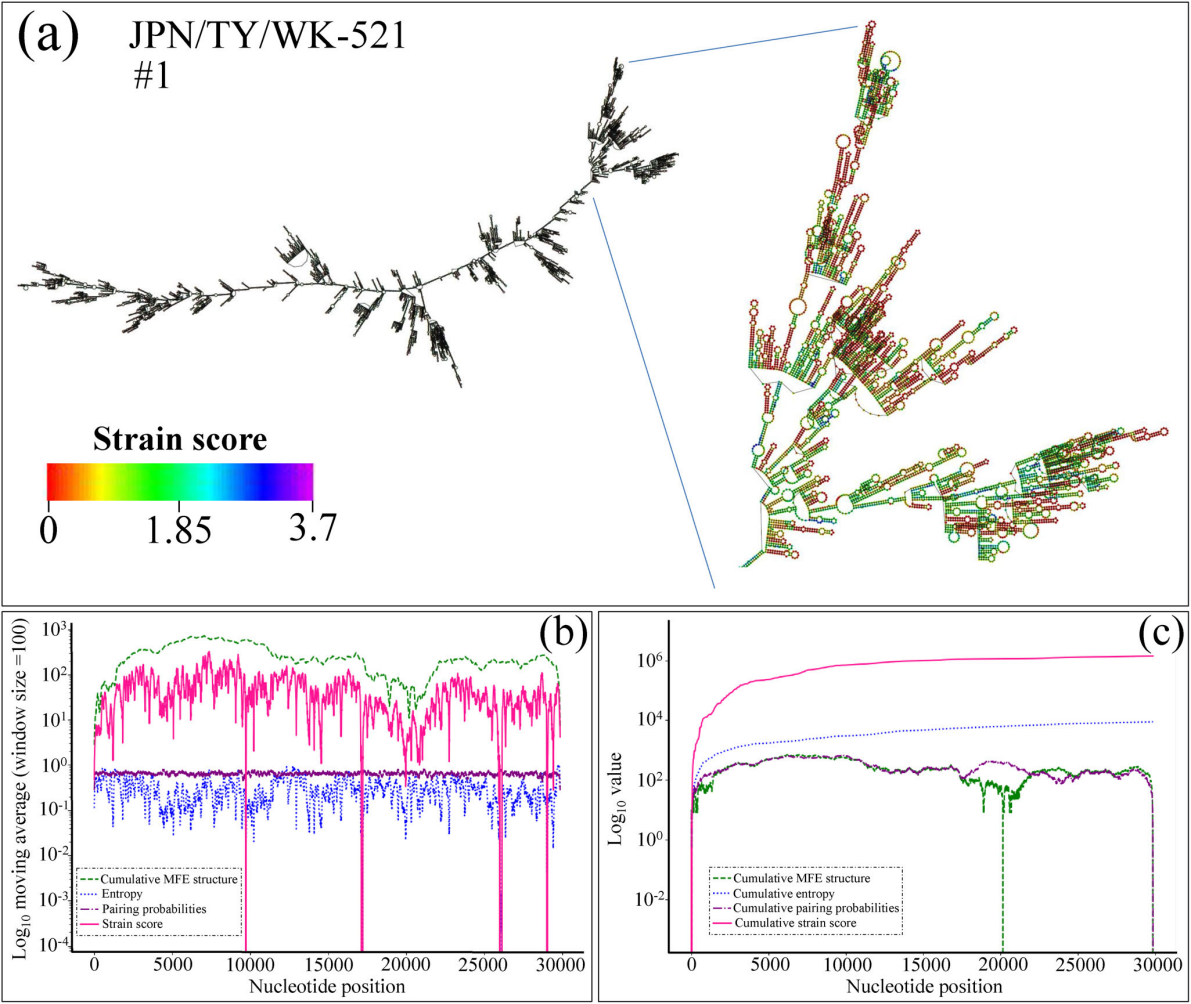
Results of the calculations via the ViennaRNA package: (a) structural map including genome strain score for the JPN/TY/WK‐521 strain (#1 Sample), (b) cMFE, BP, SE, and Strain Score, and (c) their cumulative values as a function of nucleotide position.

**FIGURE 11:**
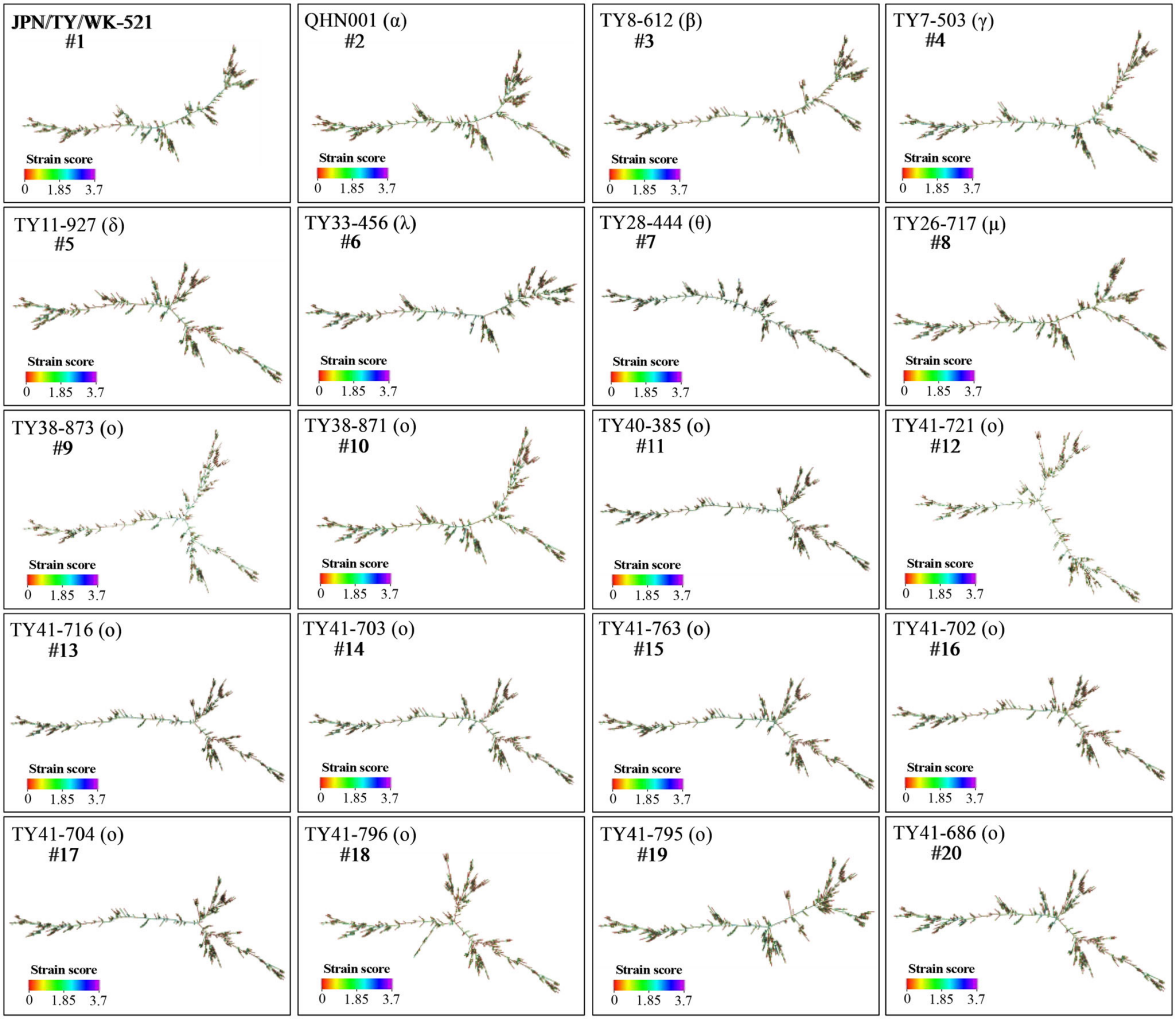
RNA secondary structures computed via the ViennaRNA package for all the studied strains (cf. labels in inset).

**TABLE 3:** List of the salient parameters used in exploring correlations between Raman signatures and structural elements, and to build up algorithms that link secondary structures to selected Raman spectroscopic features.

Label	Variant	Number of hairpin loops	Stem length	Number of stem blocks	Number of bulge loops	Total strain score
#1	JPN/TY/WK‐521	592 (3.9%)	9806 (0.53%)	3903 (0.47%)	312 (2.05%)	1422317.88 (−15.1%)
#2	QHN001 (α)	586 (2.8%)	9738 (−0.16%)	3872 (−0.33%)	305 (−0.24%)	1789798.62 (6.9%)
#3	TY8‐612 (β)	585 (2.6%)	9764 (0.10%)	3887 (−0.06%)	313 (2.38%)	1535428.14 (−8.3%)
#4	TY7‐503 (γ)	583 (2.3%)	9724 (−0.30%)	3865 (−0.51%)	304 (−0.56%)	2281518.085 (36.3%)
#5	TY11‐927 (δ)	571 (0.2%)	9745 (−0.09%)	3880 (−0.12%)	305 (−0.24%)	1121681.967 (−33.0%)
#6	TY33‐456 (λ)	575 (0.9%)	9712 (−0.42%)	3866 (−0.48%)	297 (−2.85%)	1846830.78 (10.3%)
#7	TY28‐444 (θ)	590 (3.5%)	9730 (−0.24%)	3871 (−0.35%)	300 (−1.87%)	2363936.3 (41.2%)
#8	TY26‐717 (µ)	574 (0.7%)	9782 (0.29%)	3897 (0.32%)	299 (−2.20%)	1677795.522 (0.2%)
#9	TY38‐873 (ο)	574 (0.7%)	9776 (0.23%)	3897 (0.32%)	308 (0.75%)	1918894.968 (14.6%)
#10	TY38‐871 (ο)	576 (1.1%)	9787 (0.34%)	3894 (0.24%)	311 (1.73%)	2120627.088 (26.6%)
#11	TY40‐385 (ο)	553 (−3.0%)	9739 (−0.15%)	3879 (−0.15%)	308 (0.75%)	1685866.624 (0.7%)
#12	TY41‐721 (ο)	566 (−0.7%)	9791 (0.38%)	3902 (0.45%)	309 (1.07%)	1713784.033 (2.4%)
#13	TY41‐716 (ο)	550 (−3.5%)	9757 (0.03%)	3888 (0.45%)	307 (0.42%)	1682358.366 (0.5%)
#14	TY41‐703 (ο)	560 (−1.8%)	9746 (−0.08%)	3886 (0.03%)	308 (0.75%)	1516377.764 (−9.4%)
#15	TY41‐763 (ο)	560 (−1.8%)	9742 (−0.12%)	3885 (0.01%)	307 (0.42%)	1475250.82 (−11.9%)
#16	TY41‐702 (ο)	552 (−3.2%)	9744 (−0.10%)	3882 (−0.07%)	303 (−0.89%)	1431437.1 (−14.5%)
#17	TY41‐704 (ο)	556 (−2.5%)	9746 (−0.08%)	3881 (−0.10%)	310 (1.40%)	1601196.575 (−4.4%)
#18	TY41‐796 (ο)	554 (−2.8%)	9756 (0.02%)	3889 (0.11%)	306 (0.09%)	1317738.975 (−21.3%)
#19	TY41‐795 (ο)	575 (0.9%)	9762 (0.08%)	3891 (0.16%)	307 (0.42%)	1640747.776 (−2.0%)
#20	TY41‐686 (ο)	568 (−0.4%)	9730 (−0.24%)	3879 (−0.15%)	301 (−1.54%)	1344448.8 (−19.7%)

## 4 Discussion

### 4.1 Impact of Secondary Structures on the Raman Spectrum of RNA

The overall secondary structure of RNA plays essential roles in encoding RNA regulatory fate and function and can be described by specifying both amounts and kind of chain folding present in local regions [52]. The contributions to the Raman spectrum of stems, hairpin loops, pseudoknots, bulge loops, internal loops, and branch loops (cf. Figure 9) significantly influence the Raman spectrum of RNA due to changes in base stacking, inter‐base hydrogen bonding, and backbone conformational strain. Such contributions can be overlapping, competitive, and quite differentiated, because any of them alters vibrational modes of RNA nucleotides and sugar‐phosphate backbone in distinct and peculiar ways. However, precise accounts of such effects on the Raman spectrum of specific viral RNA are yet unknown.

In stems, namely, in the presence of double‐stranded regions, base pairing and stacking are expected to lead to an intensification of Raman peaks associated with A‐form RNA helices: enhanced guanine and cytosine peaks (due to Watson‐Crick pairing) and stronger phosphate signals (due to enhanced backbone rigidity). Base stacking can also enhance C═C and C═N stretching modes, namely, signals in the 1300–1550 cm^−1^ spectral region. Hairpins disrupt base stacking at the loop, but retain it in the stem, causing shifts in both base and ribofuranosyl sugar ring vibrations; unpaired bases comprised in the hairpin loops might thus show distinctive Raman signals as compared to those in the stem, particularly in C═O (1700–1800 cm^−1^) stretching and N−H bending (1350–1450 cm^−1^) regions. This explains the significant multiplication and high variability in Raman sub‐bands observed in the two above zones (cf. Figure 1). Pseudoknots involve non‐canonical base pairing, which results in Raman signatures of non‐Watson‐Crick interactions. Such complex stacking interactions may introduce additional peaks in the fingerprint region (1200–1600 cm^−1^), while enhancing backbone‐stretching vibrations due to structural tension. Bulges, which involve one or more unpaired bases protruding from a stem (cf. Figure 9), cause local distortion of the backbone, thus altering phosphate vibrations. Their presence could thus produce base‐specific Raman shifts (depending on the identity of the bulged nucleotide) and reduced intensity in stacking‐related peaks. On the other hand, internal loops, which arise from two mismatched sequences and disrupt regular base stacking, might lead to Raman signals similar to the case of bulge loops but more pronounced due to their greater structural asymmetry. Accordingly, peaks corresponding to N−H and C═O vibrations in bases are expected to shift due to changes in hydrogen bonding. Finally, branch loops (also referred to as multibranched junctions), which involve multiple helices radiating from a central junction, lead to significant structural heterogeneity and cause a mix of Raman features from helical stems, loops, and bulges. An increased structural flexibility can cause band broadening and shifting of phosphate backbone‐related peaks.

In summary, the main takeaway notion from the above descriptions is that each secondary structure modulates the Raman spectrum uniquely with base‐stacking effects increasing Raman intensity and loop flexibility broadening or shifting peaks. Among a variety of spectral variations, pseudoknots are also expected to introduce new peaks due to unconventional hydrogen bonding, while bulge and internal loops to cause loss of intensity in structured base‐pairing regions. Such complexity, while posing a formidable challenge in its rationalization, also gives a real chance to differentiate viral RNAs through their secondary structures by tailoring operative classification tasks (e.g., algorithms of artificial intelligence trained to classify RNA spectroscopic inputs into predefined categories).

### 4.2 Raman Fingerprints of Genome Length and RNA Backbone Deformation

Previous studies by various authors have linked several Raman features to RNA secondary structure [31, 38, 41, 42, 44]. According to those studies, main spectroscopic parameters should relate to: (i) cumulative fractions of bases in single‐, double‐, and triple‐stranded regions, (ii) percentage of dimers and trimers for each base paired to its Watson‐Crick complement in double‐stranded regions, (iii) percentage of each base in single‐stranded regions, (iv) percentage of total bases in ordered single‐stranded configurations, and (v) percentage of ribofuranose‐phosphate groups in ordered configurations. In the present study, building upon the above‐cited previous literature, we attempted to screen specific spectral characteristics that should directly link to genome length and secondary structure of RNA strands in SARS‐CoV‐2 variants/subvariants.

A relative intensity plot of C−O/C−C stretching in ribofuranosyl sugar rings (seen at ∼1045 cm^−1^) as a function of percent base divergence with respect to base content averaged over all variants/subvariants (cf. Table 1) is shown in Figure 12a based on the high‐resolution spectra given in Figure 4. As also seen in Table 1, all variants/subvariants successive to the strain initially isolated in Japan (JPN/TY/WK‐521) showed a reduction in genome length. From the plot in Figure 12a, it is seen that all 20 viral strains obeyed an apparently linear dependence, the longer the genome the stronger the ribose ring signals. Similar attempts were made for signals involving the phosphodiester region of the RNA backbone: P−O−P stretching at 807 cm^−1^ (Figure 12b) and C3’−O−P bending at 432 cm^−1^ (Figure 12c) based on high spectrally resolved spectra in Figures 3 and 5, respectively. The above‐mentioned ring and backbone vibrations are schematically shown in Figure 2a. While yet obeying a direct dependence on genome length, the two latter plots display enhanced data scatter, particularly significant for the P−O−P stretching band at 807 cm^−1^ (cf. Figure 12b). This trend indicates that additional factors affect the vibrational behavior of the RNA backbone in addition to its intrinsic length. As discussed in the previous section, RNA molecules are capable to adopt multiple folds and undergo significant bending/stretching deformations due to repeat combinations of strongly bent and kinked structures [45, 48]. These circumstances could significantly affect RNA vibrational characteristics and complicate their interpretations. As discussed in detail by Gong et al. [53], the 2’‐hydroxyl group of the ribofuranosyl ring (cf. broken squares in Figure 2a) is quite unaffected by nucleotide constraining induced by base pairing [54, 55]. One could then generalize this finding and suggest that the relative intensities of vibrational modes involved with the entire ribofuranosyl ring, as the ∼1045 cm^−1^ signal monitored here, are the most reliable Raman fingerprints of genome length. On the other hand, bent and kinked RNA regions could directly affect the vibrational response of phosphodiester backbone and disrupt any direct link between Raman relative intensity and genome length. The O−P−O stretching and bending behavior should obviously be the Raman modes most affected by a bend/kink morphology, as confirmed by the plots in Figure 12b,c, in which the dependence between genome length and signal intensity becomes more scattered. In other words, phosphodiester backbone signals are much more sensitive to the local vibrational environment as compared to deoxyribose bands. Additional discussion on RNA backbone signals will be given in the next section.

**FIGURE 12:**
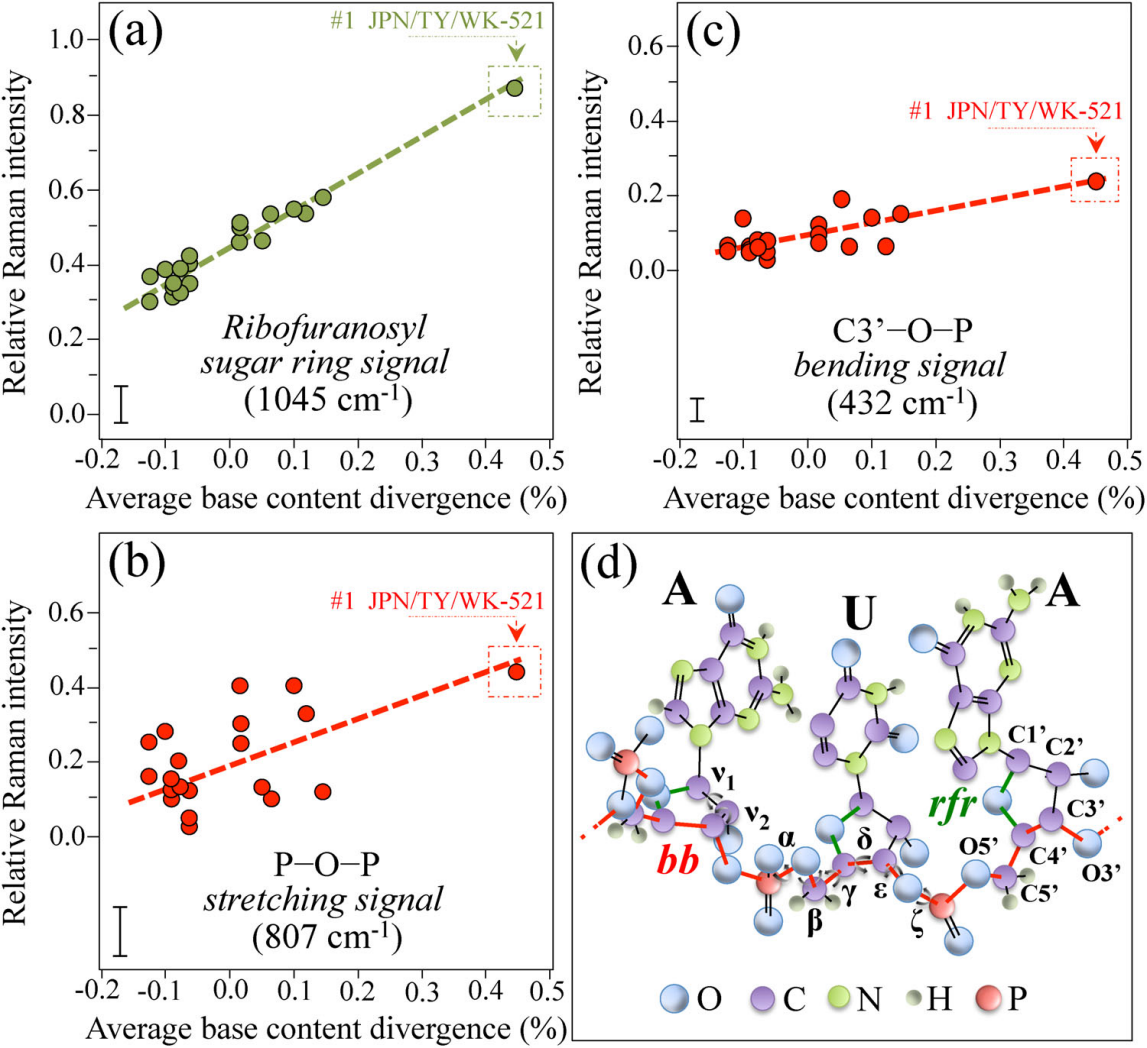
(a) Relative intensity plot of 1045 cm^−1^ C−O/C−C stretching signal in ribofuranosyl sugar rings (cf. Figure 2a) as a function of percent base divergence with respect to average base content (cf. Table 1); and, relative intensity plots for signals involving the phosphodiester region of the RNA backbone: (b) P−O−P stretching, which is seen at 807 cm^−1^, and (c) C3’−O−P bending at 432 cm^−1^. Plots are based on high‐resolution spectra in Figures 3, 4, 5. In (d), an explanatory draft is shown, which defines bending and torsion of the RNA backbone architecture according to six dihedral angles (α (P–O5’), β (O5’–C5’), γ   (C5’–C4’), δ (C4’–C3’), ε (C3’–O3’), and ζ (O3’–P)) and two additional dihedral angles on the ribofuranosyl sugar ring (ν_1_ (C2’–C1’) and ν_2_ (C3’–C2’). The draft shows that the C1’–O and C4’–O bonds are not directly related to torsional or bending displacements, while ν_1_ (C2’–C1’) and ν_2_ (C3’–C2’) only affect 2’/3’‐endo conformational switch but do not directly strain the ring. Average standard deviations for each data set are indicated at the left bottom of each plot in (a)∼(c). Abbreviations are the same as those given in Figure 1.

Figure 12d shows an explanatory draft, which offers an interpretation of the Raman data shown in Figure 12a–c. According to Dawson et al. [56], bending and torsion of the RNA backbone architecture can be described according to six dihedral angles, namely, α (P–O5’), β (O5’–C5’), γ   (C5’–C4’), δ (C4’–C3’), ε (C3’–O3’), and ζ (O3’–P); in addition to two additional dihedral angles on the ribofuranosyl sugar ring, namely, ν_1_ (C2’–C1’) and ν_2_ (C3’–C2’), which only determine the sugar architecture (i.e., pucker 3’‐endo vs. 2’‐endo). As seen from Figure 12d, the C1’–O and C4’–O bonds are not directly related to any torsional or bending displacements, while ν_1_ (C2’–C1’) and ν_2_ (C3’–C2’) only affect 2’/3’‐endo conformational switch but do not directly strain the ring. We hypothesize that this the reason why it could be possible to retrieve reliable information on genome length from the 1045 cm^−1^ vibrational signal internal to ribofuranosyl sugar rings; the longer the chain, the more numerous the rings in it, and thus the stronger the signal. On the other hand, the six dihedral angles α, β, γ, δ, ε, and ζ are directly impacted by bending/kinking (as induced by non‐canonic base pairing); such internal backbone strains heavily scatter the dependence on genome length for signals (especially the stretching ones) they are directly involved with. Non‐canonic pairing‐induced local deformations in the RNA backbone primarily occurs because such pairings disrupt both regularity of base sequence and stacking interactions, which are essential for maintaining the stability and proper conformation of the RNA structure [57, 58]. For example, a base dimer involving two purine bases that are not typically paired together can create local “kinks” in the backbone. These mispaired bases do not follow the natural helical twist, forcing the RNA to bend or stretch in that region to accommodate the irregularity. Note that base stacking interactions contribute to the helical structure of RNA by minimizing the exposure of the hydrophobic faces of the bases to the solvent. Since dimers and trimers interfere with these stacking interactions (e.g., through charge repulsion between adjacent nitrogenous rings in neighboring purines) [59], adjacent bases might not align properly for optimal stacking, and this misalignment can cause the backbone to flex or bend at that point, creating local deformation (i.e., the above‐mentioned kinks) [60]. In other words, the RNA adjusts its conformation in order to accommodate the disrupted stacking arrangement. According to molecular dynamics computations performed by Taghavi and Yildirim [49], the RNA AUUCU repeat exhibits the most extreme bending and kinked conformations. As a general pattern in all bent structures, collapse of the major groove width is the mechanical trigger for bending, which in turn is the direct consequence of a base‐pair step parameter alteration and is accompanied by overextension of the RNA repeats due to groove‐width widening and undertwisting. Another well‐characterized example of RNA‐induced kinks is the kink‐turn (k‐turn) or GA motif (cf. Figure S1). K‐turns are asymmetric internal loops that introduce sharp bends in the RNA backbone, typically around 60 degrees [61, 62]. We explored the genomic sequences for all present SARS‐CoV‐2 variants/subvariants looking for AUUCU repeats and k‐turns motifs (cf. also Figures S1 and S2). As a result, RNA AUUCU loci varied only slightly between 57 and 59. Similarly, the number of k‐turn loci fluctuated by only ∼0.88% (1598–1612) among all investigated variants/subvariants. Accordingly, neither the AUUCU repeat nor the k‐turn motif alone could be directly invoked to justify the wide scatter of Raman intensity data observed for the stretching of phosphodiester backbone chain (as shown in Figure 12b). In summary, differences in Raman spectra of SARS‐CoV‐2 RNAs did not point to any specific base‐stacking architectural feature to define RNA backbone deformation. However, high spectrally resolved Raman assessments of RNA enabled us to locate selected vibrational modes (e.g., C−O/C−C stretching vibrations internal to ribofuranosyl sugar rings) that feel dimers/trimers‐induced backbone deformations to a significantly lower extent, and thus directly link to genome length.

Fractional areal intensities of Raman ring breathing signals in purine and pyrimidine base structures at ∼724 cm^−1^ (adenine), ∼778 cm^−1^ (cytosine), ∼672 cm^−1^ (guanine), and ∼791 cm^−1^ (uracil) are plotted in Figure 13 for all variants/subvariants (cf. labels in inset) in comparison with the respective base fractions computed by genomic analyses (cf. Table 1). Fractions are given in percents of the total base Raman signal area and fractional counts in the RNA strands, respectively. The comparison shows that phase fractions computed by Raman and genome approaches greatly differed, but also revealed some repetitive pattern in such diversity. Any attempt to directly relate the detected fractional differences to the presence of specific patterns of dimeric or trimeric structures (as shown in Figures S1 and S2, respectively) failed, suggesting that differences in Raman cross‐sections are the main responsible for the observed fractional mismatches. Figure 14a–d report the percent difference in fractional contributions of the four RNA bases (cf. labels in inset) as computed from the areal fraction of their Raman signals and their fractional presences in the genomic sequence (referred to as *R‐g* fractional difference), as a function of the number of base divergence (in percents) with respect to average base content. As seen, unlike backbone‐ and ribofuranose ring‐related signals (cf. Figure 12), no plot in Figure 14 showed a detectable dependence on genome length, as indeed expected from actual genomic structures (cf. Table 1). Data scatter was higher in pyrimidines as compared to purines, which hints to their higher dependence on the secondary structure of RNA strands including the influence of hyper/hypochromic effects. Raman markers for pyrimidine rings were found to systematically overestimate base fractions, while those from purine rings revealed clear underestimations (cf. Δ*_R‐g_* values in inset to each plot). Besides differences in Raman cross‐section among signals from different bases, the observed differences in Δ*_R‐g_* values could in principle also arise from the hypo‐ or hyperchromic behavior of their ring signals. For example, we recorded the 672 cm^−1^ ring signal of guanine with a negative but low Δ*_R‐g_* value (cf. Figure 14d), in agreement with a previous study showing guanine bands following a hypochromic path when involved in stable base pairing or RNA tertiary interactions [47]. Similarly, the uracil breathing mode at 791 cm^−1^, which we recorded here as a weakly hyperchromic signal (cf. Figure 14b), could increase in intensity due to stacking interactions or hydrogen bonding effects [38]. While plots in Figure 14 empirically provide a fast path to compute base fractions from Raman data, the intertwining between Raman cross‐section and hyper/hypochromism in Raman ring signals is complex and its full rationalization would require additional theoretical studies.

**FIGURE 13:**
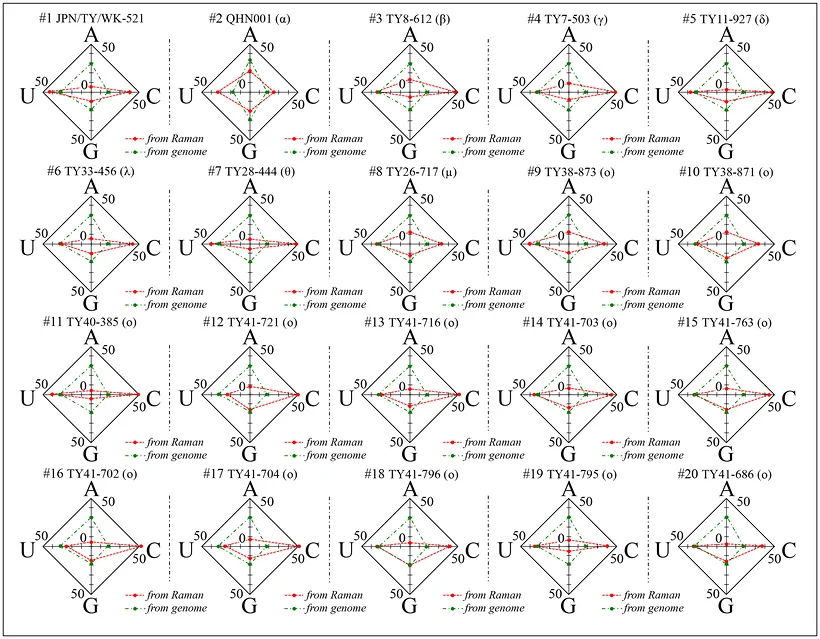
Fractional areal intensities (in %) of Raman ring breathing signals in purine and pyrimidine base structures at ∼724 cm^−1^ (adenine), ∼778 cm^−1^ (cytosine), ∼672 cm^−1^ (guanine), and ∼791 cm^−1^ (uracil) as plotted for all variants/subvariants (cf. labels in inset) in comparison with the respective base fractions (in %) computed by means of genomic analyses (cf. Table 1). Abbreviations are the same as those given in Figure 1.

**FIGURE 14:**
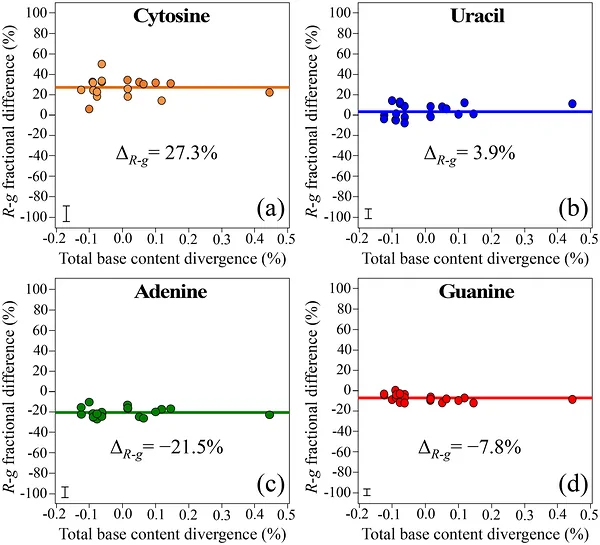
Percent difference in fractional contributions of the four RNA bases as computed from the areal fraction of their Raman signals and their fractional presences in the genomic sequence (referred to as *R‐g* fractional difference), as a function of the number of base divergence (in percents) with respect to average base content: (a) 778 cm^−1^ band (cytosine), (b) 791 cm^−1^ band (uracil), (c) 724 cm^−1^ band (adenine), and (d) 672 cm^−1^ (guanine). The average differences in fractions computed by Raman and genome analyses are shown in inset to each figure. Average standard deviations for each data set are indicated at the left bottom of each plot.

### 4.3 Raman Fingerprints of RNA Secondary Structures

Single‐stranded RNA structures tend to form intramolecular bonds between nucleotides and create patterns of secondary structures, which can uniquely characterize viral genomes [63]. As explained in Section 3.3 and summarized in Table 3, we attempted to classify the secondary structures of the SARS‐CoV‐2 RNA genomes through a series of simulations aimed at computing the fractions of secondary structures, the MFE structure, the strain score, the associated entropy, and the pairing probabilities (cf. also Figure S1 and S2).

Hairpins in viral RNA are expected to significantly affect specific Raman bands, primarily associated with base stacking, hydrogen bonding, and backbone conformations. Since hairpins consist of a stem (paired region) and a loop (unpaired region), they introduce structural heterogeneities that alter vibrational modes. In particular, RNA bending and torsional deformations as introduced by the presence of hairpins should impact on phosphodiester vibrations and, more in general, on RNA backbone modes. We screened the dependence of such bands and discovered an exponential relationship of the C3’−O−P bending signal at 432 cm^−1^ as a function of divergence of the number of hairpins from average value (Figure 15a); a trend directly arising from bending of the phosphodiester backbone in hairpin structures (Figure 15b). Note that the relative intensity of this band also showed a dependence (although more scattered) on average base content (cf. Figure 12b), hinting to an additional strain factor playing a role in this latter dependence. The Japanese ancestor variant JPN/TY/WK‐521, which is the variant with the highest number of hairpins, showed the highest relative Raman intensity at 432 cm^−1^. In other words, the exponential plot in Figure 15a shows that the 432 cm^−1^ bending signal is hyperchromic to the number of hairpins in the RNA strand. The hyperchromicity of the C3’−O−P bending signal as a function of the number of hairpins show that RNA backbone vibrations become more Raman‐active with increasing secondary structure complexity rather than just stem length. In order to explain such a spectroscopic dependence, one could, in first instance, invoke an increased structural rigidity in the backbone. Hairpin loops will introduce tight structural constraints and, in turn, a more defined geometry for the phosphodiester backbone, which likely reduces vibrational energy dissipation thus making the Raman signal both sharper and stronger. Moreover, as more hairpins form, the cumulative effect on the phosphate network could make the C3’−O−P bending mode more cooperative, leading to enhanced Raman activity. If adjacent loops/stems interact, they may further stabilize the phosphate groups, further amplifying the Raman intensity. This hypothesis is supported by the exponential nature of the plot given in Figure 15a. In other words, RNA compaction and hairpin structural organization are key drivers of Raman intensity changes in the low‐wavenumber region. This is particularly relevant in highly structured viral RNA, where hairpins do not just fold independently but may interact and form larger structural motifs. Finally, we report that a similar attempt for the P−O−P stretching band at 807 cm^−1^ failed in unfolding any relationship, thus suggesting that the relative intensity of this band obeys a multifactorial dependence of different RNA secondary structures. As a matter of fact, the symmetric phosphodiester (P–O–P) stretching 807 cm^−1^ vibration in RNA backbone is intrinsically one of the most conformation‐sensitive vibrational markers of RNA. Its intensity depends not only on genome length but also on the ensemble of phosphodiester torsion angles, backbone curvature, sugar pucker, base stacking, hydrogen‐bonding interactions, and vibrational coupling among neighboring phosphate groups [64]. Consequently, numerous secondary‐ and tertiary‐structure elements (including hairpins, bulges, internal loops, multibranch junctions, pseudoknots, and stem‐length distributions) collectively influence this Raman feature. AUUCU repeats and k‐turns should therefore be regarded only as illustrative examples of backbone perturbations rather than the dominant determinants of the observed variability.

**FIGURE 15:**
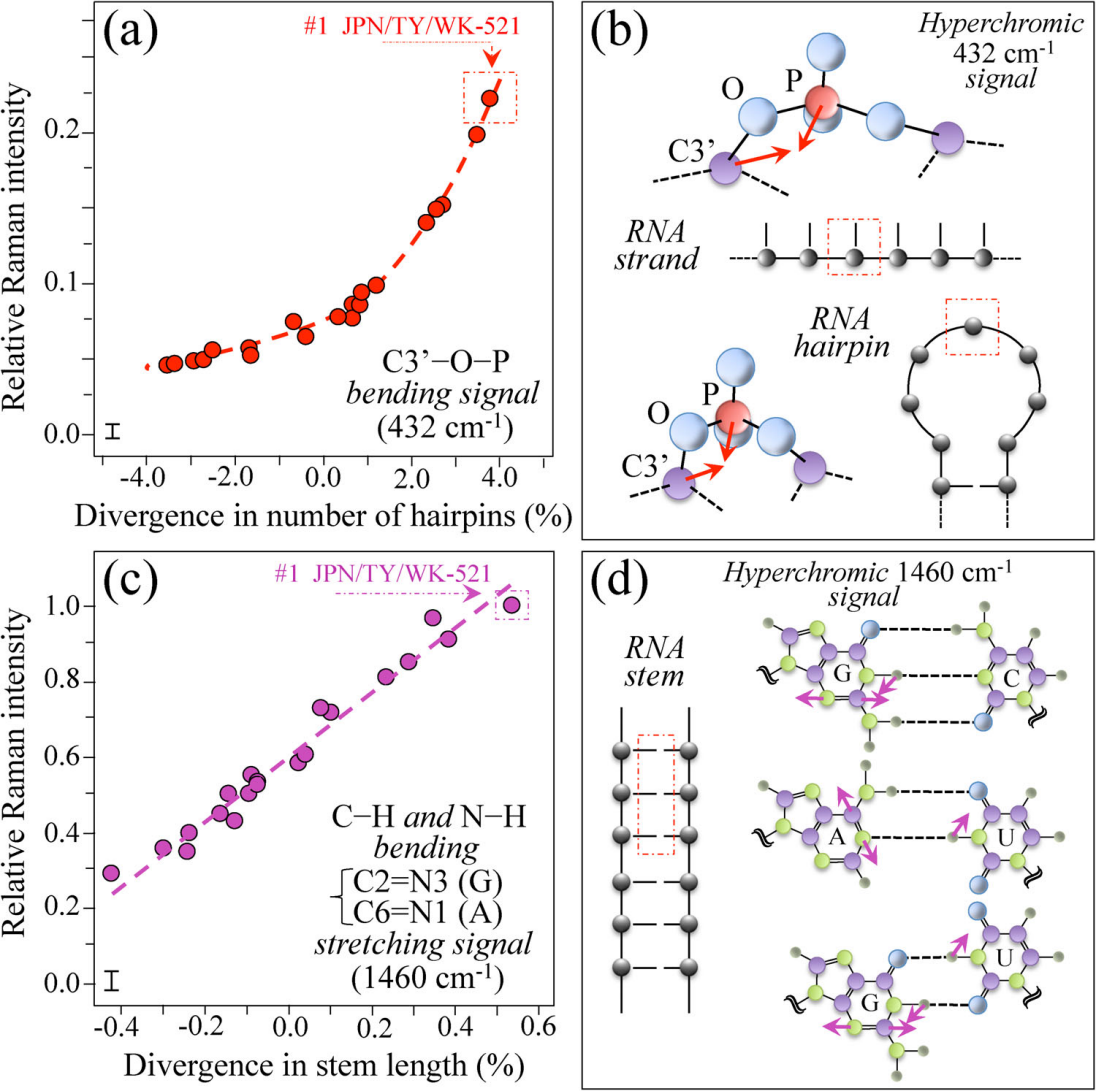
(a) Exponential hyperchromic dependence of the C3’−O−P bending signal at 432 cm^−1^ on the divergence of the number of hairpins from average value as due to (b) bending of the phosphodiester backbone in hairpin structures. In (c), the linear hyperchromic dependence of the relative intensity of the RNA signal ∼1460 cm^−1^ is given as a function of divergence from average stem‐length. The RNA signal at 1460 cm^−1^ includes multiple vibrational contributions (d), mainly associated with C═N/C═C stretching modes in guanine and adenine bases and C−H and N−H bending vibrations. Average standard deviations for each data set are indicated at the left bottom of each plot in (a) and (c).

Figure 15c shows the dependence of the relative intensity of the RNA Raman signal located at ∼1460 cm^−1^ as a function of divergence from average stem‐length value for the investigated SARS‐CoV‐2 variants/subvariants. Unlike the case of the 432 cm^−1^ signal, such dependence revealed a linear trend, the longer the stem length the stronger the Raman intensity. The RNA signal at 1460 cm^−1^ includes multiple vibrational contributions, being mainly associated with C═N/C═C stretching modes in guanine and adenine bases and C−H and N−H bending vibrations (Figure 15d) [44]. Longer stem lengths involve more base‐pair stacking and an increased number of hydrogen bonds. Such increased stacking interactions can enhance peaks in the spectral region 1300–1600 cm^−1^, which is associated with base‐ring modes of C═N/C═C stretching and bending of inter‐base hydrogen bonds. The observed hyperchromicity of the 1460 cm^−1^ Raman band with increasing stem length in viral RNA can thus be explained by changes in the molecular environment and reciprocal interactions of the nucleobases. Indeed, there could be multiple factors that concurrently contribute the observed hyperchromic trend. First, as the RNA stem length increases, an enhancement of the base‐stacking effect alters electronic interactions of the bases. This effect in turn enhances Raman intensity by modifying polarizability in these bonds. Conformational changes in RNA structure are also expected to alter dihedral angles and impact on torsional flexibility, thus leading to increases in Raman cross‐sections for specific bands. Moreover, longer stems mean more stable Watson‐Crick base pairs (A‐U, G‐C; cf. Figures 15c and S1) with an increased number of hydrogen bonds, which enhances Raman signals from C−H and N−H bending modes. In support to this argument, one could cite hyperchromicity effects reported in circular dichroism studies of RNA denaturation as induced by an increased base stacking [65, 66]. Attempts failed to perform equivalent analyses for bands of similar origins at 1340 (mainly contributed by adenine and uracil) and 1480 cm^−1^ (from guanine/cytosine and Watson‐Crick base pair‐sensitive). These bands could also be expected to show hyperchromicity with respect to stem length. However, none of these signals obeyed a clear dependence as in the case of the 1460 cm^−1^ band, which thus appeared to display a highest sensitivity to base‐stacking effects.

In summary, the 1460 cm^−1^ band uniquely tracks changes in RNA secondary‐structure organization that have been associated with viral evolutionary adaptations, including those influencing replication, translation, and immune evasion.

### 4.4 RNA Secondary Structures and the Evolution of Viral Infectivity

The secondary structure of hairpins and stems in viral RNA genome is known to play a critical role in regulating infectivity by influencing RNA stability, translation efficiency, replication, and immune evasion [67, 68]. As already demonstrated by several experimental studies [69, 70, 71], the highly conserved stem‐loop hairpin structures within the SARS‐CoV‐2 5’ untranslated region function as *cis*‐acting regulatory RNA elements that promote efficient translation initiation of the ORF1a/ORF1ab replicase polyproteins, thus supporting viral genome replication. In particular, stem‐loop 1 enables viral RNA to evade Nsp1‐mediated translational suppression, thereby ensuring synthesis of the viral replicase proteins required for establishing the replication‐transcription complex. The above‐referred studies have demonstrated that both hairpins and stems act as regulatory elements (i.e., pause sites or frameshift signals) since affecting translation of viral proteins that are essential for initiating the process and encoding viral replicase. Moreover, upon enhancing RNA stability, such structured elements can protect viral RNA from degradation by host nucleases, a more stable RNA structure meaning longer availability and more rounds of translation. According to published studies [69, 70, 72, 73, 74], stable RNA secondary structures, including stem‐loops hairpins, contribute to the persistence of the SARS‐CoV‐2 genome by reducing its susceptibility to host ribonucleases and by recruiting viral and cellular RNA‐binding proteins that stabilize the genome. The resulting increase in RNA half‐life prolongs the availability of the viral RNA as a translational template, thereby permitting additional rounds of ribosome loading, enhanced synthesis of key replicase polyproteins, and thus more efficient viral replication.

In sum, being double‐stranded regions less prone to immediate degradation as compared to single‐stranded regions, they help virions to maintain genomic integrity. The packaging signal is often structured into stem‐loops and is critical for selecting and encapsidating the full‐length genome into new virions. Mutations or structural disruptions in these elements can lead to defective packaging and reduced infectivity. In the context of viral translation efficiency, hairpins help defining binding sites and initiation points for replication. They act as “landing pads” for the translation machinery in guiding where ribosomes should link. Multiple‐structured hairpins stabilize the RNA at its 5′ end, contribute to form circular conformation favorable for replication, help to recruit the host ribosome to the translation start site, and coordinate translation initiation with the right folding landscape. In this context, the strong hyperchromicity of the C3’−O−P bending signal at 432 cm^−1^ with increasing the number of RNA hairpins (cf. Figure 15a) can be interpreted as a fingerprint for RNA stability and translation/replication efficiency, the stronger the Raman signal the higher the stability and translation/replication attitude of the specific variant.

Considering now the virus attitude to escape host immune reactions, the host innate immune system recognizes viral RNA via pattern‐recognition receptors, which are generally more sensitive to unpaired or exposed motifs (i.e., 5’ triphosphates, unstructured and long stretches of unpaired RNA). Published studies [75, 76, 77, 78] demonstrated that viral genomes, including the SARS‐CoV‐2 one, adopts an extensive network of conserved stem‐loops, hairpins, and long‐range RNA interactions that modulate its recognition by the host innate immune system. Innate immune receptors (including RIG‐I, MDA5, PKR, and the OAS family, detect viral RNA primarily through exposed double‐stranded RNA regions and characteristic RNA end structures rather than nucleotide sequence alone. By folding into compact secondary structures, the viral genome can limit the exposure of immunostimulatory duplex RNA motifs, while simultaneously recruiting viral and host RNA‐binding proteins that further influence RNA accessibility. In parallel, SARS‐CoV‐2 modifies its 5’ RNA cap through N’ and 2’‐O methylation, producing a cap structure that closely resembles host mRNA and thereby reducing activation of innate immune surveillance pathways. Together, these structural and chemical adaptations enable viral RNA to evade immune recognition while maintaining efficient translation and replication.

Single‐stranded RNA structures are more dynamic and mobile, which makes it easier for the immune system to scan and detect them. The introduction of rigid and structured hairpins and stems, within which tightly base‐pairing and structured sites hide key motifs, are expected to render viral RNA less recognizable by the immune system and thus contributes to inhibit binding to immune sensors. In addition, some structured RNA elements can also inhibit host RNA sensors directly by reducing interferon responses. According to the above reasoning, the observed hyperchromicity with stem length of the inter‐base stacking‐related signal at 1460 cm^−1^ (cf. Figure 15c) could be seen as a fingerprint for immune evasion attitude of the viral strain, the stronger the band intensity the lower the RNA flexibility and the amount of exposed single‐stranded regions, and the less immunorecognizable the RNA structure.

Figure 16 shows a Raman intensity plot (re‐scaled from 0 to 100) for the 432 and 1460 cm^−1^ bands, which, according to the above reasoning, could be assumed as indicators for translation/replication and immune escape efficiencies, respectively. The abscissa of the plot, which shows denominations of different variants are given, is in turn also a temporal sequence, since variants/subvariants are listed in the order of their successive appearances. In the context of the secondary structure contribution to virus infectivity, the ancestor variant showed the highest efficiency with respect to both above functions. Throughout the development of successive variants, the translation/replication efficiency showed a constant decrease for successively developed new variants, with the exception of the θ variant (cf. label #7 in Figure 16). This variant indeed displayed an RNA secondary structure with the closest efficiency score to that of the ancestor one. According to Rose et al. [79], the variant of concern θ contained multiple concerning mutations in the spike protein, which have indeed been linked to increased transmissibility and immune escape [80]. In general, ancestor variants of viruses are not inherently more replication‐efficient than their successive mutations, which replication efficiency typically depends on the ability to adapt to the host environment. Over time mutations could either increase or decrease the virus replication efficiency, depending on how well they are suited to host and other environmental factors. While the majority of the viruses tend to evolve in ways that enhance replication, there are indeed multiple examples of viruses whose replication efficiency has diminished with developing successive variants in trade‐offs between translation/replication and drug resistance/immunity escape. For example, in the human immunodeficiency virus (HIV), specific mutations in the reverse transcriptase or protease genes have conferred resistance to antiretroviral drugs, but also have negatively impacted replication efficiency [81, 82]. In other words, mutations required for drug resistance have reduced HIV viral fitness in the absence of drug. In the case of influenza virus, frequent mutations (e.g., antigenic drifts in surface hemagglutinin protein) to evade immunity and help the virus to persist in the population have reduced the virus replication efficiency, impaired binding to host cell receptors, and limited translation efficiency [83, 84]. Hepatitis C virus, known for its high mutation rate, has also shown loss in replication efficiency in the context of an increasing immune escape ability, this phenomenon being denominated as “viral fitness cost” [85, 86]. The balance between immune evasion, drug resistance, and replication efficiency is thus a key factor driving viral evolution. In the case of SARS‐CoV‐2 viral evolution, emergence of the Omicron variant has helped the virus evade immunity, but strongly reduced replication efficiency, a phenomenon mainly related to the development of vaccines [28, 87, 88, 89]. This is indeed confirmed in the present study, which showed an evolution of the secondary structure of viral RNA starting from the µ variant (#8) and continuing with a wave of high immune escape efficiency (i.e., high relative Raman intensity at 1460 cm^−1^; cf. Figure 16) in correspondence of the successive Omicron variants (#9∼#13). The increased transmissibility of Omicron variants, as a consequence of their better immune evasion, appears to have gained an additional (although less intense) spike in correspondence of a successive Omicron variant (#19). This trend indeed appears to be in agreement with the most recently reported SARS‐CoV‐2 evolution trends [90].

**FIGURE 16:**
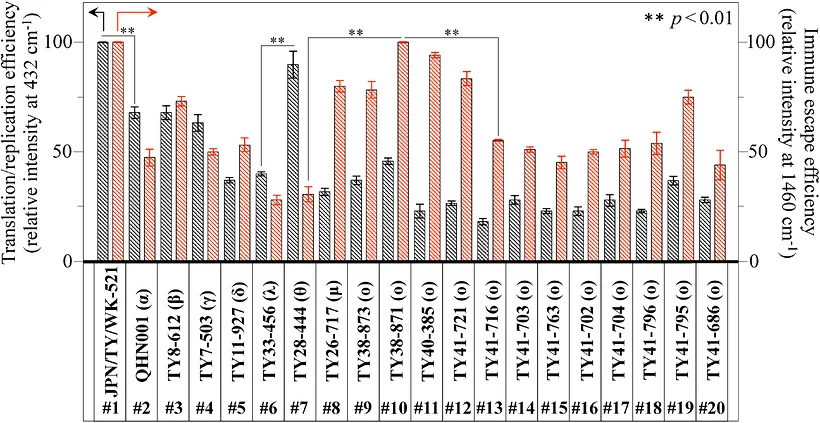
Raman intensity plot re‐scaled from 0 to 100 for the 432 and 1460 cm^−1^ bands as indicators for translation/replication and immune escape efficiencies, respectively; the plot abscissa is given as a sequel of denominations for different variants, but it also represents a temporal sequence, since variants/subvariants are listed in the order of their successive appearances. Standard deviations and statistical validations are given in inset.

In summary, mutations that disrupted hairpins and reduced stem length, as observed in the evolution of SARS‐CoV‐2 variants with time (cf. Figure 16) contributed to diminish viral translation or replication efficiency, impaired the RNA interactions required for viral functions, and promoted exposure of the RNA to host defenses. With these concepts in mind, the hyperchromicity of Raman bands at 432 and 1460 cm^−1^ could be seen as a strong indicator for RNA secondary structure characteristics directly correlated with active regulatory roles of viral infectivity.

### 4.5 Raman Genome Barcode as a Complementary Approach in Virology

Real‐time speciation and fast tracking by means of advanced Raman spectroscopic procedures could represent a significant step forward in virology. PCA‐based chemometrics, a machine learning methodology based on morphological similarities among collected spectra, is nowadays a widely used tool in biomedical spectroscopy [91, 92, 93]. In this study, we applied the PCA analyses to 20 Raman spectra (each average of 10) collected on RNA samples of different variants/subvariants of SARS‐CoV‐2 virus. However, the conventional PCA approach failed in speciating the majority of viral RNA under examination. To overcome this failure, we applied a simple Raman barcode procedure [25, 50, 51] to high spectrally resolved (average) virus spectra and succeeded in speciating all samples with high statistical reliability. We also showed that the Raman barcode is encrypting all the secondary‐structure RNA characteristics, which represented the most crucial factor of diversity among spectra collected on different variants/subvariants. Note that the proposed methodology aims at replacing (or complementing) the sequencing stage of current molecular diagnostic workflows rather than the RNA extraction stage, which remains common to essentially all RNA‐based diagnostic platforms.

A critical step in building the Raman barcode is represented by the deconvolution of the spectrum into a series of sub‐band components. As explained in Section 2.2, we used an automatic solver that summed up spectra from selected elementary molecules from to a library and fitted their sum to the experimental spectrum with preserving relative intensities, spectral positions, and full‐width at half‐maximum values for all individual sub‐bands of each elementary compound [22]. It is important to consider that each elementary spectrum in the library is itself composed of predefined Raman sub‐bands whose relative intensities, spectral positions, and full‐width‐at‐half‐maximum values are preserved throughout the optimization. Consequently, the fitting procedure does not introduce independent peak parameters at arbitrary spectral positions, but rather adjusts only the relative contributions of physically meaningful molecular components. This strategy dramatically reduces the number of adjustable degrees of freedom compared with conventional unconstrained Lorentzian‐Gaussian fitting and therefore minimizes the possibility of overfitting or non‐unique solutions, even in spectrally congested regions.

Barcodes are already used in medical procedures either to reduce the risk of human error during medication administration (e.g., overdosing patients or missed medication errors) [94] or in classifying pathogenic species (e.g., DNA barcoding of bacterial strains and yeast isolates) [95, 96, 97, 98]. DNA barcoding, which nowadays represents a global initiative for species identification, is based on sequencing short DNA sequence markers at selected genetic loci. Compared to the presently available DNA barcoding approaches, the main advantage of the Raman barcoding proposed here is the possibility of skipping DNA/RNA analyses and directly replacing them with a Raman spectrum, thus leading to a much faster identification. This will in turn enable on site identification of the pathogenic species in times solely dictated by RNA extraction from the sample. Once a complete Raman library of virus spectra is built up and the spectral deconvolution procedure fully standardized, the proposed Raman barcoding approach could directly be translated to clinical setting with all the above‐mentioned advantages. This study shows that, although based on a completely different principle, namely, the screening of secondary RNA structure rather than base sequence, the Raman approach could provide virus discriminatory criteria with the same precision of NGS, and with swiftness comparable with RT‐PCR. The present data also demonstrate that Raman spectroscopy has the potential to provide virologists with a library of spectra that can be used to unravel the makeup of unknown viral RNA. While it is not expected that conventional methods for virus detection will be supplanted by Raman‐based analyses, at least until it could be fully elaborated also from a theoretical viewpoint, Raman data contain crucial information on the secondary structure of genetic sequences, which represent a rich source of information to ascertain virus evolution.

In summary, Raman spectroscopy of viral RNAs, augmented with the computation of Raman barcodes, were found to be capable of reliably recognizing all SARS‐CoV‐2 variants/subvariants. Therefore, a specifically designed Raman instrumentation could allow virologists to rapidly identify viral RNAs in a faster way and with lower analytical costs. Crucial to this approach will be the availability of a wide library of RNA Raman spectra and their related barcodes based on a properly standardized spectral deconvolution algorithm.

### 4.6 Limitations of this Study

The present study represents a first step toward the definition of a “Raman genome” based on the vibrational fingerprints of viral RNA. Although the proposed approach successfully discriminated all twenty investigated SARS‐CoV‐2 variants and subvariants, several limitations should be acknowledged.

First, the study was conducted on a representative, yet finite, set of SARS‐CoV‐2 strains. While these included the original Japanese isolate, all major variants of concern, and a broad panel of Omicron subvariants, additional studies involving a larger number of emerging variants and independent viral isolates will be necessary to further assess the generality and robustness of the proposed Raman barcode algorithm. In particular, the ability of the method to identify newly emerging variants that differ by only a limited number of mutations remains to be systematically evaluated.

Second, the interpretation of spectral differences relies primarily on the established relationships between Raman vibrational features and RNA secondary‐structure motifs. Although the assignments proposed in this study are supported by extensive literature on nucleic acid spectroscopy and by the observed correlations with predicted RNA secondary‐structure parameters, future studies integrating Raman spectroscopy with experimentally determined RNA structural maps are expected to refine these assignments and improve our understanding of sequence‐dependent vibrational signatures.

Third, Raman spectra provide information on the average molecular organization of the extracted RNA molecules. Consequently, the present approach does not identify individual nucleotide substitutions or directly determine the primary nucleotide sequence, as achieved by next‐generation sequencing. Instead, Raman spectroscopy detects the structural consequences of sequence variations as reflected in changes in RNA conformation, nucleotide stacking, backbone geometry, hydrogen‐bonding interactions, and base‐pairing patterns. Therefore, the proposed methodology should be regarded as complementary to genomic sequencing rather than as a replacement for it.

Another limitation concerns experimental standardization. Raman spectra are influenced by instrumental configuration, excitation wavelength, spectral resolution, laser power, acquisition parameters, sample preparation, RNA purity, and spectral preprocessing procedures. Although all measurements in the present study were performed under strictly identical experimental conditions, broader inter‐laboratory validation will be required before universally applicable Raman barcodes can be established. The development of standardized acquisition protocols, automated spectral deconvolution procedures, and shared reference spectral libraries will be essential for future clinical implementation.

A further limitation concerns the standardization of the computational workflow used for Raman barcode generation. Although the present deconvolution algorithm employs a constrained machine‐learning solver based on a reference spectral library in which the relative intensities, peak positions, and full‐width‐at‐half‐maximum values of the constituent Raman bands are preserved, further validation is required to establish its robustness with respect to preprocessing variables such as baseline correction, spectral normalization, signal‐to‐noise ratio, and instrumental variability. Comprehensive inter‐laboratory validation using standardized datasets will be necessary to define universally applicable protocols for Raman barcode generation and to ensure reproducibility across different instrumentation and measurement conditions. Such methodological standardization represents an important objective of our ongoing research but lies beyond the scope of the present proof‐of‐concept study.

Note also that, in the present study, all Raman measurements were performed on purified viral RNA extracted from controlled cell‐culture supernatants. Although this experimental design allowed the intrinsic structural signatures of SARS‐CoV‐2 RNA to be investigated without interference from other biological constituents, clinical specimens such as nasopharyngeal swabs contain variable amounts of host RNA, genomic DNA, proteins, mucus, cellular debris, and microbial material. While standard RNA extraction procedures substantially reduce these contaminants, additional studies are required to determine the minimum RNA purity, RNA integrity, and viral load necessary for reliable Raman barcode generation from clinical samples. Validation across diverse specimen types and standardized extraction protocols will therefore be essential before routine clinical implementation can be considered.

Finally, although the Raman barcode approach demonstrated clear discrimination among all investigated variants, the present study was performed on purified viral RNA under controlled laboratory conditions. Additional investigations are needed to evaluate the performance of the methodology using clinical specimens containing variable RNA concentrations, partially degraded RNA, or complex biological backgrounds.

Despite these limitations, the present work establishes the proof of concept that Raman spectroscopy captures structural information encoded within viral RNA that is not readily accessible through conventional compositional analyses. The ability to rapidly classify SARS‐CoV‐2 variants according to their RNA structural fingerprints supports the concept of a “Raman genome” and provides a promising foundation for future developments in rapid viral diagnostics, molecular surveillance, and preparedness for emerging infectious diseases.

## 5 Conclusion

In this paper, we proposed a Raman spectroscopic approach to RNA diagnostics complementary to RNA sequencing, which enables a prompt and accurate classification of SARS‐CoV‐2 variants/subvariants and with potential for a conclusive identification of newly emerging ones. Upon classifying RNA Raman spectra according to the proposed Raman barcode algorithm, the presented spectroscopic approach succeeded in promptly and unambiguously differentiating 20 SARS‐CoV‐2 variants/subvariants where conventional PCA failed. All 20 RNA samples showed quite different “Raman genomes”, despite their base fractions computed from the genome database being conspicuously homogeneous. Such amplified differences in RNA Raman signals directly link to RNA secondary structures and enable viral speciation down to the subvariant level in a time frame solely due to RNA extraction (i.e., without the need of RNA sequencing).

In this first step toward a new “Raman genome” approach, differences in spectral signatures could partly be unfolded and mainly related to the secondary structure of RNA. They were related to ribose rings, phosphate linkages, and hyper/hypochromism of specific signals from RNA bases sensitive to pairing effects and other features related to the RNA secondary structure. The present results also suggest that Raman spectroscopy of viral RNA could have a role in fast screening of new variants, thus adding invaluable advantages in emergency and critical care environments. However, further advancements of the Raman technology are needed to improve its automation, to standardize spectral deconvolution algorithms, and to expand our understanding of molecular fingerprints from nitrogenous bases in different neighboring pair environments through theoretical computations and artificial intelligence algorithms. These topics are presently work in progress. Nevertheless, the present study deliberately adopted a reductionist strategy aimed at identifying physically interpretable relationships between individual Raman bands and defined RNA secondary‐structure descriptors. Although multivariate chemometric methods may improve predictive performance by simultaneously incorporating numerous correlated spectral variables, they generally provide less direct insight into the molecular origins of the observed spectral variations. Establishing these molecular‐level spectroscopic markers is considered here a fundamental prerequisite for future predictive models integrating multiple structural descriptors and machine learning approaches.

## Author Contributions

**Giuseppe Pezzotti**: conceptualization, writing – original draft, methodology, validation, visualization, writing – review editing, formal analysis, project administration, data curation, supervision, resources. **Yoshiki Yasukochi**: methodology, software, formal analysis, data curation, writing – review editing. **Gianluca Angiola**: investigation, writing – review editing, data curation. **Takaharu Ueno**: investigation, writing – review editing. **Saki Ikegami**: investigation, writing – review editing, formal analysis, data curation. **Rintaro Okawa**: investigation, writing – review editing, data curation. **Tetsuya Adachi**: investigation, writing – review editing, resources, funding acquisition. **Wenliang Zhu**: writing – review editing, methodology, supervision. **Osam Mazda**: conceptualization, funding acquisition, project administration, resources, supervision. **Alfio Grillo**: conceptualization, writing – review editing, project administration, data curation. **Koichiro Higasa**: writing – review editing, methodology, software, data curation. **Kazu Okuma**: conceptualization, writing – review editing, resources, supervision.

## Funding

This work was financially supported by Japan Society for the Promotion of Science [Grant Number JP23K09359].

## Conflicts of Interest

The authors declare no conflicts of interest.

## Supporting information




**Supporting File**: advs77679‐sup‐0001‐SuppMat.docx.

## Data Availability

The Raman spectra data that support the findings of this study are openly available in “figshare” at doi.org/10.6084/m9.figshare.30050821. And the data of the nucleotide sequence that support the findings of this study are available in GISAID at https://gisaid.org, accession numbers shown in the text.
