# Supplementary material for: Raman Analysis of RNA Nucleotide Strands From SARS‐CoV‐2 Variants and Subvariants: A Step Forward in the Definition of “Raman Genome”
Source: Adv Sci (Weinh). 2026 Sep 30:e77679. Online ahead of print. doi: 10.1002/advs.77679 (PMC13627450; doi:10.1002/advs.77679)
Supplement: Supplementary file 1 [file ADVS-9999-e77679-s001.docx]

**Raman Analysis of RNA Nucleotide Strands**

**from SARS-CoV-2 Variants and Subvariants: A Step Forward in the Definition of “Raman genome”**

Giuseppe Pezzotti, Yoshiki Yasukochi, Gianluca Angiola, Takaharu Ueno, Saki Ikegami, Rintaro Okawa, Tetsuya Adachi, Wenliang Zhu, Osam Mazda, Alfio Grillo, Koichiro Higasa, and Kazu Okuma

**Supplementary Information**

**Figure S1:** (a) Frequencies of neighboring dimers averaged over all the investigated variants/subvariants; in (b)~(u), similar plots are given for each single variant/subvariant investigated (cf. labels in inset). Computations were made by means of the ViennaRNA software package. Abbreviations are the same as those given in Figure 1 of the paper.

**Figure S2:** (a) Frequencies of neighboring trimers averaged over all the investigated variants/subvariants; in (b), similar plots are given for each single variant/subvariant investigated (cf. labels in inset). Computations were made by means of the ViennaRNA software package. Abbreviations are the same as those given in Figure 1 of the paper.
